# Supplementary material for: Testing the convergent validity, domain generality, and temporal stability of selected measures of people’s tendency to explore
Source: Nat Commun. 2024 Sep 4;15:7721. doi: 10.1038/s41467-024-51685-z (PMC11375013; doi:10.1038/s41467-024-51685-z)
Supplement: Supplementary file 1 — Supplementary Information [file 41467_2024_51685_MOESM1_ESM.pdf]

Software for Data Collection and Analyses

Data collection was done using the oTree software platform (https://www.otree.org/). Data cleaning and analyses were done in R version 4.2.3, using the following packages (version number in parentheses): DescTools (0.99.48;); corrgram (1.14); psych (2.2.9, and 2.4.6.26); corrplot (0.92); plyr (1.8.8); TOSTER (0.6.0); ltm (1.2.0); pacman (0.5.1); lavaan (0.6-18); MVN (5.9); EGAnet (2.0.6); paran (1.5.2); parallel (4.2.3); semTools (0.5-6); and tictoc (1.2).

Information Sheet and Consent Form

Screenshot of the information sheet participants read before beginning the study:

Information sheet

Dear participant,

Thank you for taking part in our study. This study about individual differences in decision making. The basic details of the study are as follows:

- It will take approximately **60 minutes** to complete the study.
- The study consists of 5 tasks followed by some questionnaires.
- **Important!** Each task has written instructions followed by comprehension questions that need to be answered correctly before continuing with the task. If you get these comprehension questions for any one task wrong more than 5 times, you will not be able to earn any further bonuses.
- You will be paid **£6.00** to complete the study (show-up fee).
- You will also be paid a **bonus payment** based on your performance. We expect that, on average, participants will earn a bonus totaling about **£5.00** in addition to the show-up fee. You can significantly increase your bonus payment by trying to maximize points earned in every task.
- Please allow up to **3 working days** to receive your payment.

At the end of the study, there will be a link that will redirect you to the Prolific.ac website. If you have questions please contact [redacted]

Thank you very much for your attention and participation!

Please press "Next".

Screenshot of consent form:

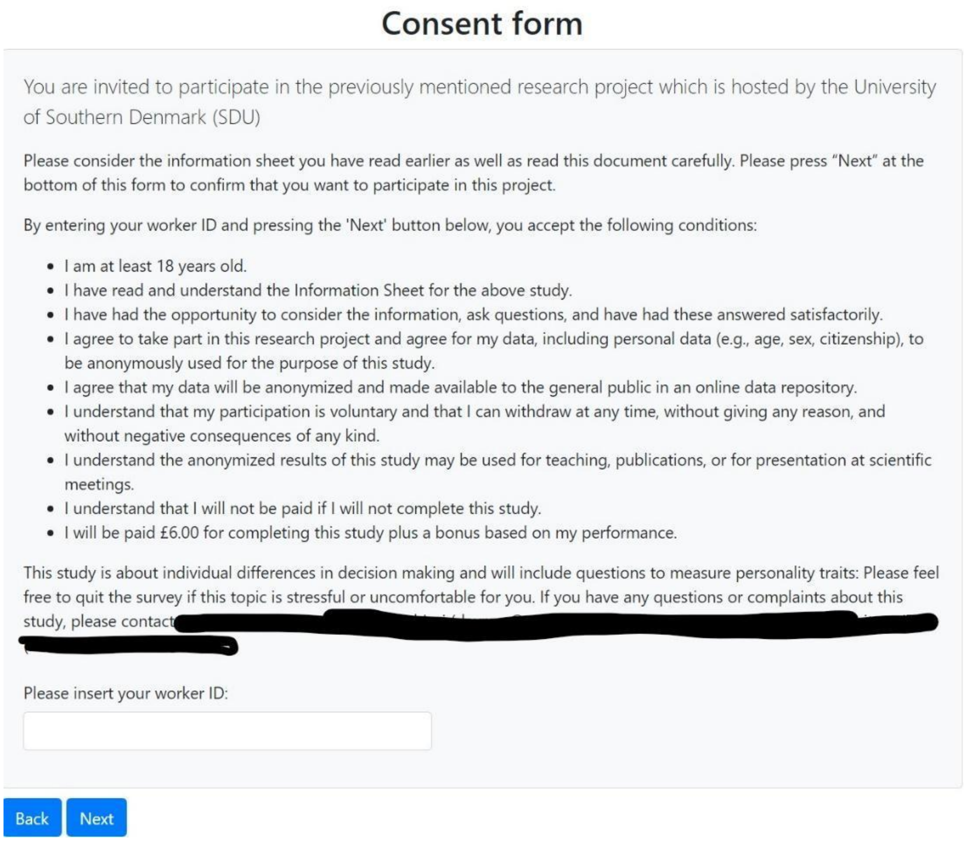
Consent form

You are invited to participate in the previously mentioned research project which is hosted by the University of Southern Denmark (SDU)

Please consider the information sheet you have read earlier as well as read this document carefully. Please press "Next" at the bottom of this form to confirm that you want to participate in this project.

By entering your worker ID and pressing the 'Next' button below, you accept the following conditions:

- I am at least 18 years old.
- I have read and understand the Information Sheet for the above study.
- I have had the opportunity to consider the information, ask questions, and have had these answered satisfactorily.
- I agree to take part in this research project and agree for my data, including personal data (e.g., age, sex, citizenship), to be anonymously used for the purpose of this study.
- I agree that my data will be anonymized and made available to the general public in an online data repository.
- I understand that my participation is voluntary and that I can withdraw at any time, without giving any reason, and without negative consequences of any kind.
- I understand the anonymized results of this study may be used for teaching, publications, or for presentation at scientific meetings.
- I understand that I will not be paid if I will not complete this study.
- I will be paid £6.00 for completing this study plus a bonus based on my performance.

This study is about individual differences in decision making and will include questions to measure personality traits: Please feel free to quit the survey if this topic is stressful or uncomfortable for you. If you have any questions or complaints about this study, please contact [redacted]

Please insert your worker ID:

Back Next

Details and Instructions for Behavioural Tasks

Following the consent form, participants begin completing either the behavioural tasks or the self-reports (independently randomized for each participant). For each task, after reading the instructions, participants need to answer 3 comprehension check questions correctly before beginning the task. If they get a question wrong a pop-up message informs them that they answered incorrectly and they are redirected to the instructions page. Participants who do not answer all of the questions correctly after 6 attempts for any one task will be automatically moved onto the next task.

Multi-Armed Bandit

Task background. In bandit problems[1,2], participants repeatedly choose among two or more options whose expected value (and, in general, distribution parameters) is unknown and can only be inferred through experience. After each choice, participants learn the reward from the selected option. If on the one hand exploratory choices allow the decision maker to gain more information about the value of alternatives (and thus form more accurate expectations about these values), on the other hand they imply the indirect cost of missing the opportunity of selecting the alternative with larger expected value. Although often computationally challenging, it is possible to find an optimal solution to some of these problems.[2] Experimental studies of behaviour in bandit problems have highlighted robust tendencies such as that of over-exploitation of poor alternatives and under-exploration of good ones[3], the tendency to mostly rely on the outcomes

very recently experienced[4], and the so-called “hot-stove effect”, according to which experience of a bad outcome dramatically lowers the probability that the corresponding option is selected again[5].

Instructions to participants. Participants viewed the following instructions:

Task Instructions

In this task, five buttons will be displayed on the screen, and on each trial you have to select one of the five buttons by clicking on it. Each time you select a button, you earn some points. Buttons differ from one another in the average points that they yield. There will be 20 trials in the practice block and 40 in each of the remaining 4 blocks.

Note that after each click the system needs about one second to process your entry.

The average payout from each button remains the same within each block, but may change from one block to the next.

Earnings

At the end of each block, you'll get feedback about how many points you earned in that block. The first block will be for practice, but the points you earn from the remaining 4 blocks will be used to determine your bonus payment for this task. For this task, you will be paid a bonus of 1 pence (£0.01) per 100 points.

Next

Comprehension checks. The comprehension check questions were (correct answer have an asterix *):

1) How many buttons can you sample from in this task? [This is an open text response with acceptable answers being “5” or a case insensitive “five”]

2) After you click on a button, you will be presented with:

a) The points from the selected button*

b) The points you would have gotten by selecting the other buttons

c) The colour of the button

3) How many trials are in each block (not including the practice block)?

a) 10

b) 40*

c) 45

Task parameters. The five buttons presented on the screen (the options to choose from) are associated to a normal distribution with mean μ_i, $i = 1, ...5$, and variance $\sigma^2 = 25$. Thus, on each trial, clicking on button i, $i = 1, \ldots, 5$, reveals a non-negative payout drawn from a normal distribution with mean μ_i and variance $\sigma^2 = 25$. At the beginning of each block, the means μ_i are initialized as follows: One mean is randomly drawn from the interval [43, 48], two from the interval [49, 54], and two from the interval [55, 60]. The means are randomly allocated to

buttons and the possible payoffs are bounded within the interval [0, 100], although this truncation minimally affects payoffs (when the mean is equal to 43 or 60, respectively the smallest and largest possible value, the probability of observing a payoff outside the interval [0, 100] is negligible).

Screenshot of the multi-armed bandit:

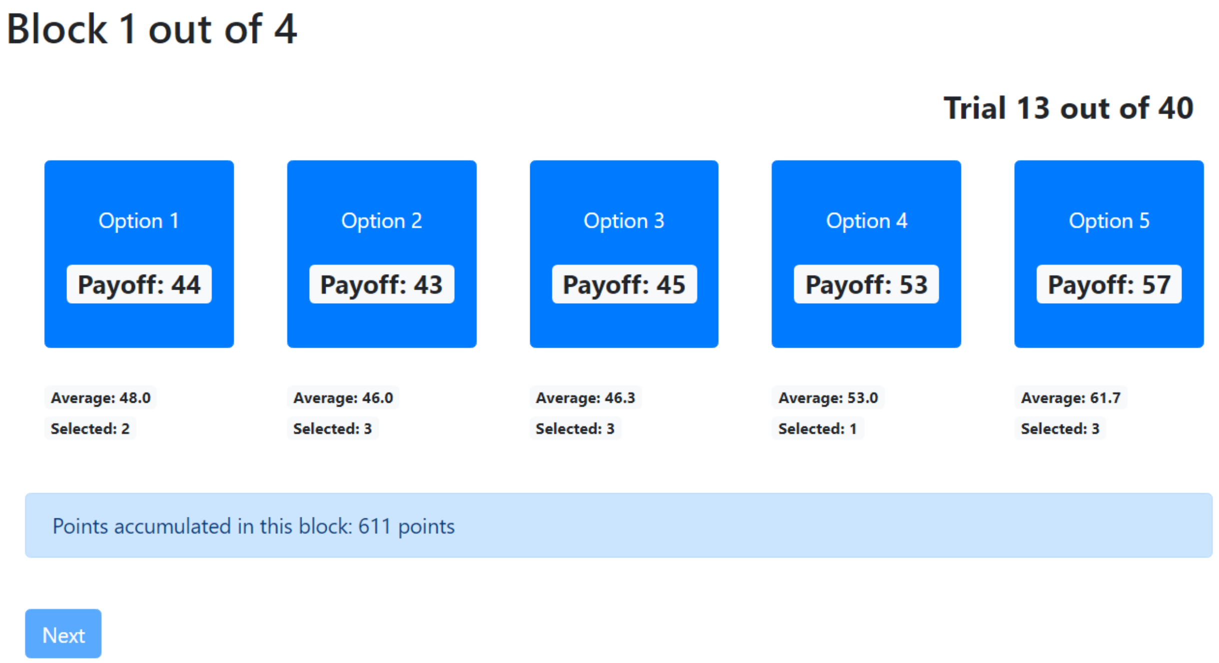

Alien Game

Task background. The alien game is a task that allows for the examination of combinatorial multi-attribute decision-making in environments with different degrees of complexity.[6] Several implementations and variations of the alien game exist.[6–8]

Instructions to participants. Participants viewed the following instructions:

Task Instructions

In this task you create pictures by selecting and/or deselecting symbols presented in a row across 10 columns (see screenshot below). There are 10 different symbols and each row of symbols represents a picture. An alien from outer space likes to buy such pictures and it will pay different amounts for different pictures with different symbols, but you don't know what the alien likes and how the interconnectedness between the different symbols influences the payoff. Each block consists of 10 trials (a first example picture and its payoff are already displayed), and in every block you will face a different alien.

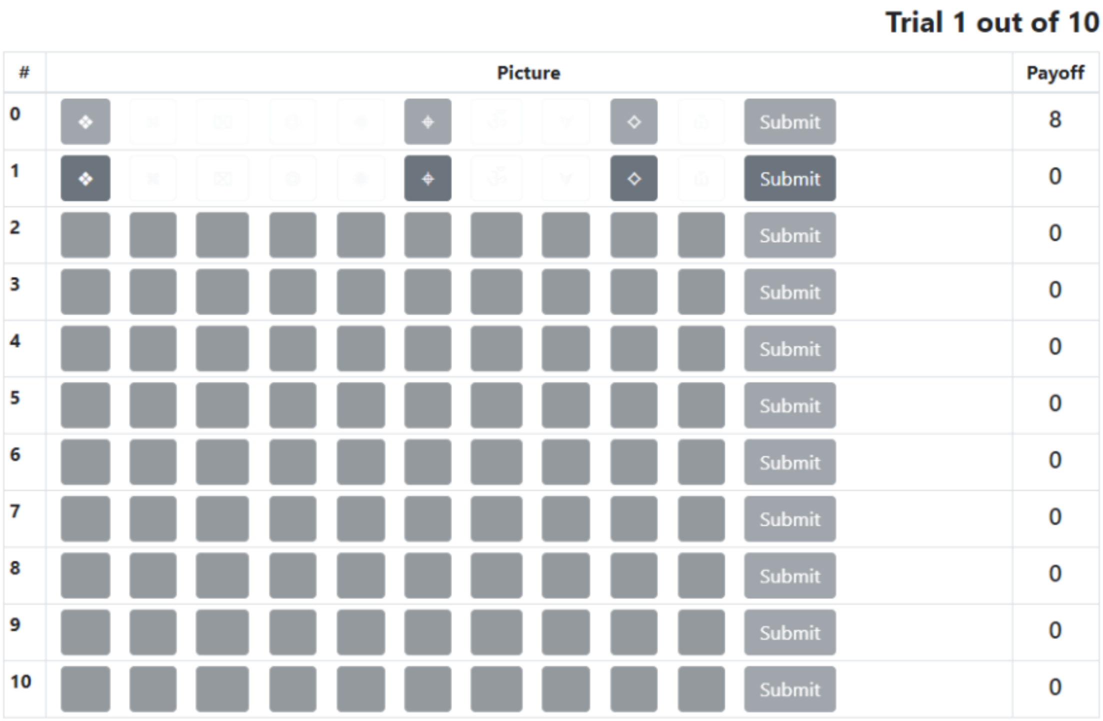

On each trial you can combine symbols any way you like to make pictures: you can select or deselect as many symbols as you wish (0-10), but you must make a decision for every symbol in every trial as the alien considers the entire combination of selected and not-selected symbols. When you're ready you can click on the "Submit" button and the price will then be shown under the heading "Payoff" and the trial is completed.

If you submit the same picture in the same block, you'll be paid the same price. At the end of each block, the alien buys all of the pictures you created in that block and pays you the accumulated price. The value of the pictures will change from one block to the next.

Earnings

The first block will be for practice, but the points you earn from the remaining 3 blocks will be used to determine your bonus payment for this task. For this task, you will be paid a bonus of 2 pence (£0.02) per 10 points.

Next

Comprehension checks. The comprehension check questions were (correct answers have an asterix *):

1) What creature is buying the pictures? [This is an open text response with acceptable answers being case insensitive “alien” or “aliens”]

2) How many symbols can you change from one trial to the next?

a) 0-10*

b) 1

c) 1-5

3) How many pictures is the creature willing to buy?

a) The alien buys all pictures and pays a random price

b) The alien buys one picture. It is the one with the highest price

c) The alien buys all pictures and pays the accumulated price*

Task parameters: We used parameters based on Billinger et al.[6,9] with four standard NK landscapes,[10,11] which are for every participant randomly allocated to the practice block and one for each of the three main blocks. All landscapes had the same parameters with N=10 and K=3, offering 2^{10}=1024 alternative combinations in a medium-low complex environment in which three out of ten unknown attributes are interdependent with other attributes. The landscapes have one global maximum, one global minimum, 16 local peaks, and 16 local valleys. The different landscapes are all normalized and the payoff associated with each possible combination of attributes does not change within the same block, but differs across blocks. The payoffs of the various combinations are all values between ~0.25 and 1, and they are all multiplied by the same uneven multiplier (24.3) to obscure the range of possible outcomes. When subjects start with the Alien Game, a first combination is already displayed with a payoff in round one. This combination resembles the least performing combination in a given landscape (following Billinger et al.[6,9]) and it puts all subjects in the same starting position (i.e. the global minimum in each landscape). It is important to note is that participants are not made aware of the starting combination’s location in the landscape. Participants also do not have any information concerning the global maximum, the number of peaks or valleys, or the magnitude of changes possible, and they are forced to engage in trial-and-error search and exploration to identify high-valued combinations within each landscape. The NK landscapes used for the study are available on request; the layout of the experiment and the design of the buttons / attributes can be found in the screenshot below.

Screenshot of the alien game:

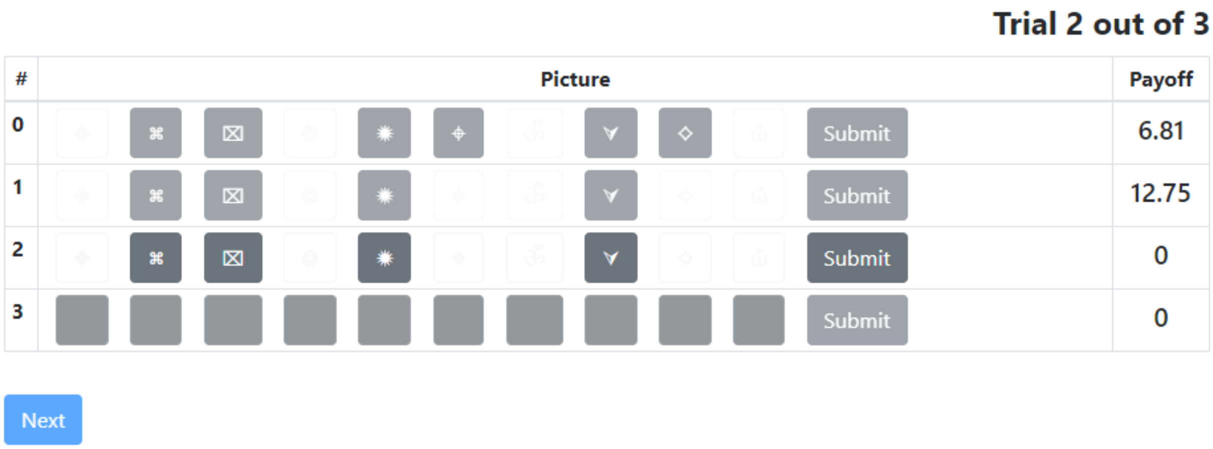

The payoff values are displayed with two decimals, which means that individuals observe payoffs with three or four digits. For the calculation of the search distance (or Hamming distance) we rely on the bitstring comparison of the current configuration and the best-performing prior configuration within the same block. In the very rare event that the best performing payoff occurs more than once (within a block), we use the configuration with the most recent payoff for the calculation of the search distance. Search distance is a count variable that takes values between zero and ten. It can be observed from trial two onwards, since the comparison of two configurations is not possible in trial one.

Optional Stopping Task with Recall

Task background. Optional stopping tasks were originally developed in statistics and were quickly adopted by economists as they can be used to model consumer search or search for better prices.[1,12] The optimal solution to the problem is to define a stopping threshold that optimally trades off the value acquired from sampling new options with the cost of search and then selecting the first option searched with a value higher than this threshold.[1] In our task the threshold for each level of cost, 0.05, 0.1, 0.2, and 0.4, are 9.01, 8.59, 8, and 7.17, respectively. Previous work on the task has suggested substantial interindividual variability in the number of options that different people search, with a majority of people searching fewer options than the optimal strategy, but a sizable minority searching more.[13] Here we implement a version of the task where the participants look at the options sequentially and they can go back (recall) only to the best option encountered so far.[13,14]

Instructions to participants. Participants viewed the following instructions:

Task Instructions

In each block of this task, you will search for rewards among 20 different boxes. You can open a box by clicking on it, which reveals how much it's worth. Each box has a randomly determined value between 0 and 10. However, every time you open a box you pay a cost ranging from 0.05 to 0.4 points depending on the block that you are in.

After opening a box, you can either settle on the highest value among the opened boxes, thereby finishing that block, or you can keep opening more boxes (up to a maximum of 20).

The costs for opening boxes, and the rewards from each box, changes from one block to the next.

Earnings

At the end of each block you will receive the highest value you have discovered so far minus the total cost of opening boxes. The first block will be for practice, but the earnings of the remaining 8 blocks will be added up and used to calculate your bonus. For this task, you will be paid a bonus of 2 pence (£0.02) per point.

Next

Comprehension checks. The comprehension check questions were (correct answers have an asterix *):

1) How many options or boxes do you choose from? [This is an open text response with acceptable answers being "20" or a case insensitive "twenty"]

2) The cost of search is:

a) 0.5 points per box

b) 0.1 points per box

c) from 0.05 to 0.4 points per box*

3) The value behind the boxes could be:

a) Any value between 10 and 20

b) Any value between 0 and 100

c) Any value between 0 and 10*

Task parameters. Full details of the task parameters are presented in the main text.

Screenshot of the optional stopping task:

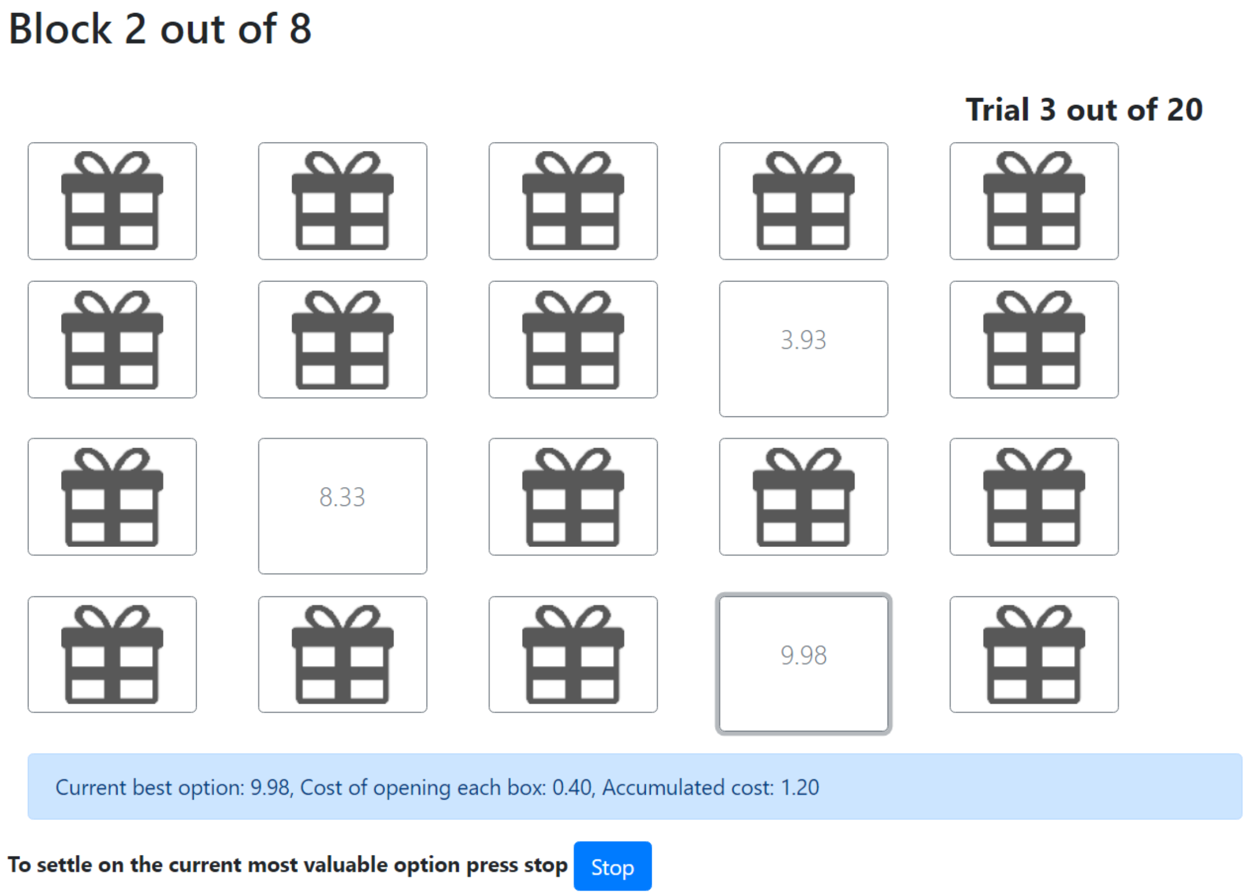

Sampling Paradigm

Task background. The sampling paradigm, or decisions from experience task[15], was designed to investigate the effect of rare events and people's reliance on information search in risky choices. The buttons have different payout probabilities, typically involving one button that is riskier than the other.

Instructions to participants. Participants view the following instructions verbatim:

Task Instructions

In this task you'll see two buttons on your screen. Each button has some probability of paying out a certain number of points and zero points otherwise. Your task in each block is to decide which button you prefer to be paid out from.

Before deciding, you can sample from each button by clicking on it to reveal what it would have paid out if you had chosen it. You must sample from each button at least once before making your final decision, but you can keep sampling from the two buttons (up to a maximum of 100 times) until you're confident enough to decide.

You can make a final choice by toggling the grey button below the two options and then pressing "Next". You can move the toggle to the left or right to choose the option on the left or right, respectively. Once you choose a button, you'll see how many points you earned from it and the block will end.

Note that after each click the system needs about one second to process your entry.

The average payout from each button remains the same within each block, but may change from one block to the next.

Earnings

The first block will be for practice, but the points you earn from the remaining 5 blocks will be used to determine your bonus payment for this task. You'll be paid a bonus of 5 pence (£0.05) per point.

Next

Comprehension checks. The comprehension check questions were (correct answers have an asterix *):

1) How many buttons do you choose from? [This is an open text response with acceptable answers being "2", or a case insensitive "two"]

2) What is the minimum number of times you have to sample from each button?

   a) Three

   b) One*

   c) Two

3) How do you learn about the payouts from a button?

   a) Click on "choose" and then select the button

   b) Pay 1 point to look

   c) Click on the button to sample from it*

Task parameters. We used the following payout distributions taken from past research[16]:

Training block: one button pays 10 with probability 0.1 (zero otherwise), other button pays 1 with probability 1;

Paying blocks: one button pays 4 with probability 0.8, other button pays 3 with probability 1;

one button pays 16 with probability 0.2, other button pays 3 with probability 1;

one button pays 4 with probability 0.2, other button pays 3 with probability 0.25;

one button pays 32 with probability 0.1, other button pays 3 with probability 1;

one button pays 10 with probability 0.9, other button pays 9 with probability 1.

The paying blocks are presented to participants in random order and the buttons within each block are also randomized.

A screenshot of the sampling paradigm:

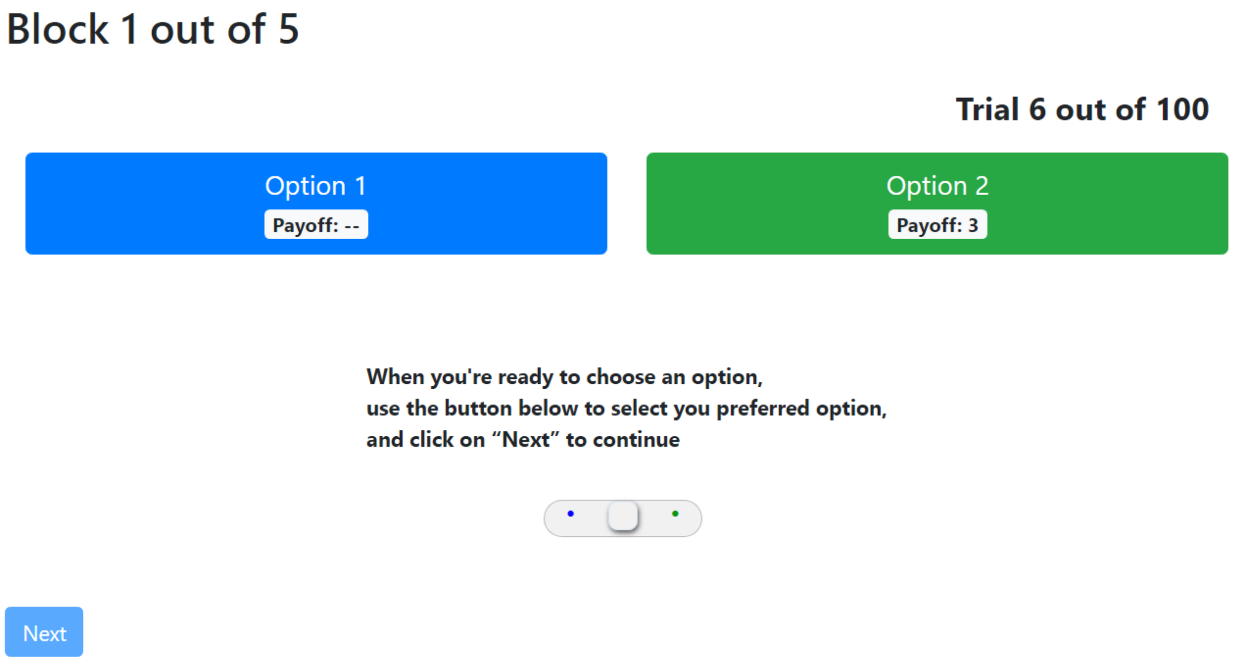

Observe or Bet Task

Task background. The observe or bet task[17] was designed to isolate information search (or exploration) from reward gathering (or exploitation) to investigate people's information seeking behaviour and deviations from the optimal strategy in such tasks.

Instructions to participants. Participants view the following instructions:

Task Instructions

In this task, you'll see one light bulb on your screen and three buttons. On each trial you will click on one of the three buttons and then the light will turn on either blue or red but you won't always see what colour it turns on. You'll only see what colour the light turns on if you choose the "observe" button. The light will have a higher probability of turning one colour than the other and this probability will remain the same throughout each block of trials but will change from one block to the next.

Before each trial you can choose one of three actions by pressing one of the three buttons on the screen: "guess blue", "guess red", or "observe". If you choose to observe then you will be shown which colour the light turns on, but you won't get any points. If you guess either blue or red, then your guess will be recorded, but you won't be shown whether the guess was correct (i.e., you won't see the light turn on).

For each correct guess you get 1 point and for each incorrect guess you lose 1 point. At the end of each block you'll get feedback about how many points you earned in that block.

Earnings

The first block will be for practice with only 25 trials, and the following 2 blocks will have 50 trials each and will be used to determine your bonus payment. You'll be paid a bonus of 4 pence (£0.04) per point.

Next

Comprehension checks. The comprehension check questions were (correct answers have an asterix *):

1) How many light bulbs are in this task? [This is an open text response with acceptable answers being "1", or a case insensitive "one"]

2) What happens if you choose to "observe"?

   a) You see how much each option is worth

   b) You will see what colour the light turns on*

   c) You will not see the light turn on

3) What happens if you guess either "blue" or "red"?

   a) The game stops

   b) You will see what colour the light turns on

   c) You will not see the light turn on*

Task parameters. The buttons had the following probabilities of turning on in any given trial (whether the blue or red light turned on with the higher probability is randomized across participants):

Training block: light turns on one colour with probability .55, otherwise it turns on the other colour;

Paying blocks: light turns on one colour with probability .60, otherwise it turns on the other colour;

light turns on one colour with probability .70, otherwise it turns on the other colour.

The paying blocks are presented to participants in random order.

Screenshot of the observe or bet task:

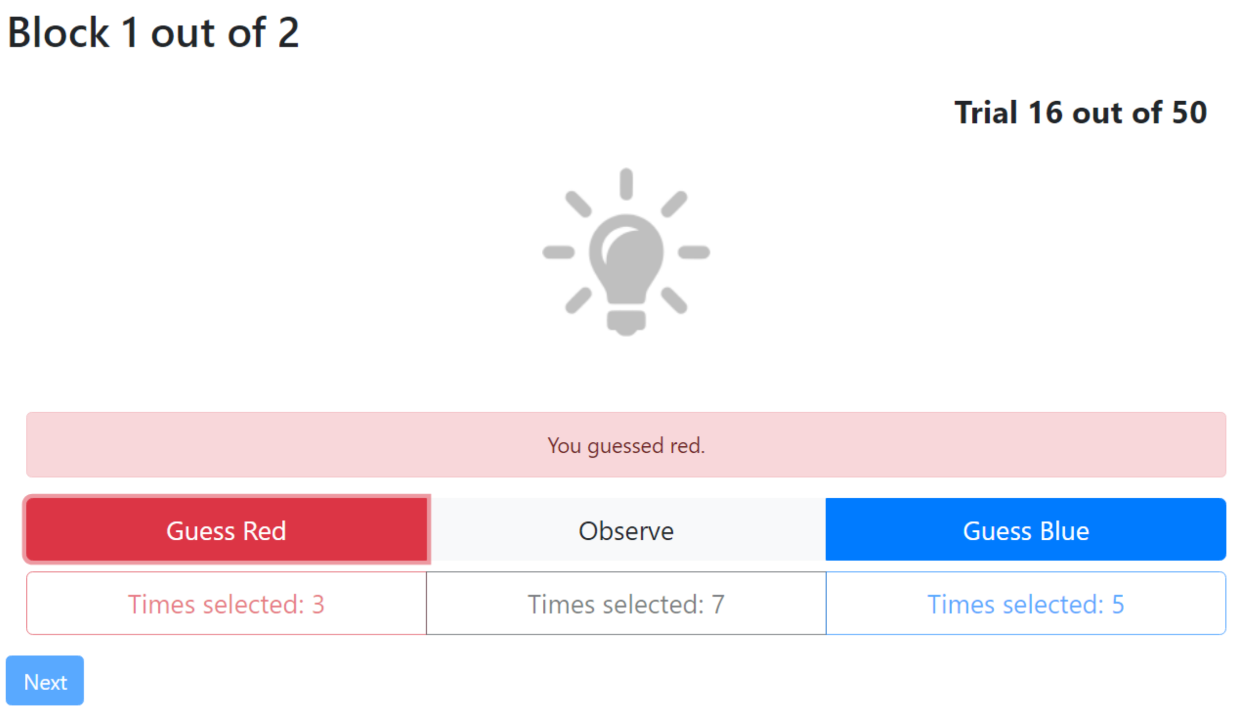

Details of Self-Report Scales

Exploration Scale

The self-reported exploration scale is one facet of the Curiosity and Exploration Inventory II.[18] The first version of this inventory[19] has been widely adopted by psychologists. A central facet of curiosity is exploration (also called "stretching"), defined as "actively seeking opportunities for new information and experiences" ([18], p. 989). The scale has 5 items: "I actively seek as much information as I can in new situations", "I am at my best when doing something that is complex or challenging", "I view challenging situations as an opportunity to grow and learn", "I am always looking for experiences that challenge how I think about myself and the world", and "I frequently seek out opportunities to challenge myself and grow as a person". These statements are rated on the following scale: 1= very slightly or not at all, 2 = a little, 3 = moderately, 4 = quite a bit, 5 = extremely. The tendency to explore is measured by the average rating across all of these items.

Maximizing and Satisficing

Schwartz et al.[20] expanded Simon's[21,22] concept of maximizing and satisficing by proposing that certain individuals have the tendency to maximize when making decisions whereas other individuals have a tendency to satisfice. Re-conceptualizing maximizing as a stable individual trait that varied between people, Schwartz et al.[20] developed a self-report scale to measure maximizing. This scale consists of three factors: decision difficulty, alternative search, and high standards. They proposed that individuals who score high on a maximization scale would engage in more exploration, such as searching through more options. The original scale developed by Schwartz et al.[20] has been criticized and revised.[23–27] However, the relevant alternative search subscales from many of these revised scales tend to be outdated, including questions to do with channel surfing on TV, how often people change the channel on the car radio, writing letters, and renting videos (things people these days tend not to be engaged with). Indeed, Misuraca and Fasolo[28] have argued that maximization scales need to be updated to reflect what people do in modern times.

Maximization tendency scale. Highhouse et al.[23] criticized multidimensional measures of maximization and defined maximization as the "pursuit of the best option" (p. 365) which implies a general tendency to search for the best alternative and mirrors the framings in some of the behavioural tasks (e.g. optional stopping task). Highhouse et al.'s measure of maximization (the maximization tendency scale) should therefore converge with the other measures of exploration, both behavioural and self-report. Harman et al.[29] found a positive though not statistically significant relationship between the maximization tendency scale and number of samples drawn from the sampling paradigm (r = .11, N = 123), and Rim et al.[25] found a statistically nonsignificant negative relationship (r = -.05, N = 124). Again, however, it is unclear whether the issue of validity is to do with the self-report scale or the behavioural measure of exploration in the sampling paradigm. Thus, to test convergence of this self-report measure of exploration, the maximization tendency scale, we include it in our study.[25]

The maximization tendency scale has 9 items: "No matter what it takes, I always try to choose the best thing", "I don't like having to settle for 'good enough'", "I am a maximizer", "No matter

what I do, I have the highest standards for myself", "I will wait for the best option, no matter how long it takes", "I never settle for second best", "I am uncomfortable making decisions before I know all of my options", "Whenever I'm faced with a choice, I try to imagine what all the other possibilities are, even ones that aren't present at the moment", and "I never settle". All items are rated on a scale from 1 = *strongly disagree*, to 5 = *strongly agree*; the other points are not labelled. The average rating across all items provides a measure of the tendency to explore, with higher numbers representing a greater tendency.

Alternative search (maximization inventory). Recent research has not found statistically significant relationships between the number of samples in the sampling paradigm (a behavioural measure of exploration) and scores on the alternative search scale; *r*s = -.11 in study 1, and .04 and .08 in study 2).[29] Importantly, these studies cannot establish whether the maximization scale's alternative search lacked validity or the sampling paradigm. Moreover, Rim et al.[25] found that alternative search and decision difficulty were positively correlated with exploration as measured by number of draws in a sampling paradigm (p. 576). Thus, research is needed using different behavioural and self-report measures of exploration for more complete evidence regarding construct validity of the various measures and the psychometric properties of a potential domain-general exploration construct. We will therefore test convergence of the alternative search subscale from Turner et al.[26] with the other measures of exploration.

The alternative search subscale from the Maximization Inventory has 12 items: "I can't come to a decision unless I have carefully considered all of my options", "I take time to read the whole menu when dining out", "I will continue shopping for an item until it reaches all of my criteria", "I usually continue to search for an item until it reaches my expectations", "When shopping, I plan on spending a lot of time looking for something", "When shopping, if I can't find exactly what I'm looking for, I will continue to search for it", "I find myself going to many different stores/online shops before finding the thing I want", "When shopping for something, I don't mind spending several hours looking for it", "I take the time to consider all alternatives before making a decision", "When I see something that I want, I always try to find the best deal before purchasing it", "If a store/online shop doesn't have exactly what I'm shopping for, then I will go somewhere else", and "I just won't make a decision until I am comfortable with the process". The items that involve shopping were adapted to include online shopping. The items are rated from 1 = *strongly disagree*, to 6 = *strongly agree*; the other points are not labelled. The average rating across all items gives a measure of the tendency to explore (higher ratings represent a greater tendency).

Past Research on Convergent Validity of Self-Reports with Behaviour

One line of research found no reliable relationship between self-reports and exploratory behaviour in the sampling paradigm (Pearson's *r* ranged from -.18 to .23)[29]; whereas another line of research found a positive correlation between the Alternative Search subscale of the Maximization Inventory and exploratory behaviour in the sampling paradigm (r = .32; Study 4)[25]. Therefore, evidence for convergence between self-report and behavioural measures of exploration tendencies is limited, and the results are inconclusive.

Computational Analysis of Exploration Measures in the Bandit Task

We ran simulations to understand how the two proposed measures of explorative behaviour in the multi-armed bandit task, i.e., the switch rate and best-reply-complement rate are related to each other. To this end, we simulated the behaviour of artificial agents acting repeatedly in a choice environment that closely mirrors our experimental settings (i.e., same number and parameterization of available options and same total number of trials). We modeled agents' choice behaviour via the softmax rule (equation 1), according to which the probability $p_{i,t}$ with which an agent selects option *i* at trial *t* is defined as:

$$p_{i,t} = \frac{exp\ (\underline{\pi}_{i,t} \cdot \tau)}{\sum_{j=1}^{5} exp\ (\underline{\pi}_{j,t} \cdot \tau)},\ i = 1, \dots, 5 \qquad \text{(equation 1)}$$

where $\underline{\pi}_{i,t}$ is the average obtained reward from action *i* at time *t*, and τ is the unique free parameter of the model (ranging in [0, +∞)). The τ parameter tunes an agent's propensity to exploit the action with the largest average reward: When $\tau = 0$, then the agent behaves randomly, selecting each action with the same probability, irrespective of the obtained average reward; on the contrary, as $\tau \rightarrow +\infty$, the agent deterministically best replies to the option with the largest average reward. For the simulations, we initialize average rewards $\underline{\pi}_{i,0}$ at trial $t = 0$ with one random draw from the relevant distribution.

We simulated choice behaviour of a population of 10,000 agents under different values of τ (see Table S0 below), and each time computed for each agent the two behavioural measures of exploration we use in our study (i.e., the frequency of switches among options, and the complement to 1 of the frequency of selections of the choice with the largest average reward). In essence, we wanted to know for a given level of τ, how much agreement there would be between the switch-rate and the complement to 1 of the best-reply rate.

Varying the value of τ has a non-monotonic effect on the Pearson's product-moment correlation coefficient between the two measures. Whereas for mid-values of τ the correlation is large (in absolute value) and negative, when τ assumes larger values or approaches zero, the correlation decreases. This result indicates that depending on the propensity to explore all throughout the experiment (determined by τ), these two measures appear to capture different behavioural tendencies.

In sum, although intuitively measuring the same construct, we find that the correlation between the two measures of explorative behaviour in the multi-armed bandit is consistently negative and varies considerably in absolute value with the softmax parameter that determines the level of exploration all throughout the experiment. For this reason, we decided to include both in our analysis plan.

Table S0.

Results of simulation in MAB

τ values	Estimated correlation	Significance
0.001	-0.012	0.240511
0.0025	-0.036	0.0003
0.005	-0.097	<.0001
0.01	-0.209	<.0001
0.02	-0.530	<.0001
0.03	-0.734	<.0001
0.05	-0.882	<.0001
0.1	-0.940	<.0001
0.25	-0.948	<.0001
0.5	-0.914	<.0001
1	-0.834	<.0001
2	-0.709	<.0001
4	-0.560	<.0001
8	-0.457	<.0001

Note. Simulation results, with 10,000 agents.

Results from Pilot Data

The pilot studies produced a total of 38 complete observations across all measures except the measures derived from the alien game. Due to a software programming error, we ended up with 20 observations for the alien game. Therefore, for each pairwise correlation presented in Figures S1 and S2, below, the sample size is 38, except for the correlations involving the measures from the alien game where the sample size is 20. Figure S1 presents the pairwise correlations in numerical form with the uncorrected 95% confidence intervals, as well as in the form of circles, the size and opaqueness of shading both reflecting the magnitude of the correlation. Figure S2 presents the one-sided *p*-values for each of the pairwise correlations, with circles present for pairwise correlations for which the *p*-value is below the corrected alpha of .005. We examined the internal consistency of each behavioural measure of exploration. We examined the correlation for each behavioural measure of exploration across the different incentivised blocks of each task. We report these here (all reliability tests are two-tailed).

The bandit task has two measures of exploration, the switch rate and the complement of the best reply rate (reported as the "complement" from here on). When correlating the first 2 blocks with the final 2 blocks, the switch rate ($r_{(36)}$ = .77, $CI_{95\%}$[.60, .88], $p < .0001$) and complement ($r_{(36)}$ = .81, $CI_{95\%}$[.66, .90], $p < .0001$) both showed good consistency. We also found good internal consistency when correlating odd blocks (i.e., blocks 1 and 3) with the even blocks (i.e., blocks 2 and 4), for the switch rate ($r_{(36)}$ = .90, $CI_{95\%}$[.81, .95], $p < .0001$) and the complement ($r_{(36)}$ = .91, $CI_{95\%}$[.83, .95], $p < .0001$).

For the alien game, the hamming distance showed good internal consistency whether we correlated block 1 with blocks 2 and 3 ($r_{(18)}$ = .75, $CI_{95\%}$[.46, .90], $p = .0001$), blocks 1 and 2 with block 3 ($r_{(18)}$ = .80, $CI_{95\%}$[.55, .92], $p < .0001$), or blocks 1 and 3 with block 2 ($r_{(18)}$ = .75, $CI_{95\%}$[.46, .90], $p = .0001$). The measure of active search in the alien game also showed internal consistency, though somewhat lower than the Hamming distance, when we correlated block 1 with blocks 2 and 3 ($r_{(18)}$ = .39, $CI_{95\%}$[-.07, .71], $p < .0906$), blocks 1 and 2 with block 3 ($r_{(18)}$ = .49, $CI_{95\%}$[.06, .77], $p = .0280$), and blocks 1 and 3 with block 2 ($r_{(18)}$ = .59, $CI_{95\%}$[.20, .82], $p = .0061$). Although one of the correlations was not statistically significant for active search, this could simply be due to the small sample size in the alien game (i.e., 20). Moreover, the confidence intervals indicate that the internal correlations observed are not inconsistent with substantially larger relationships (i.e., r = .71).

For the optional stopping task, internal consistency was good when correlating the first 4 blocks with the last 4 blocks ($r_{(36)}$ = .67, $CI_{95\%}$[.45, .82], $p < .0001$), or odd blocks with even blocks ($r_{(36)}$ = .61, $CI_{95\%}$[.37, .78], $p < .0001$).

In the sampling paradigm, internal consistency was also good when correlating the first 2 blocks with the last 3 blocks ($r_{(36)}$ = .50, $CI_{95\%}$[.22, .71], $p = .0013$), first 3 blocks with the last 2 blocks

($r_{(36)}$ = .53, $CI_{95\%}$[.25, .73], p = .0007), or odd numbered blocks with even numbered ones ($r_{(36)}$ = .65, $CI_{95\%}$[.42, .80], p < .0001).

The observe or bet task also showed good internal consistency in terms of the correlation between the number of observe trials in the first block with the number of observe trials in the second block ($r_{(36)}$ = .60, $CI_{95\%}$[.35, .77], p < .0001).

Results from Simulations on Test-Retest Reliability

The behavioural tasks included in the study have a stochastic component such that by luck of the draw some people will sometimes get a high (or low) payoff on earlier trials. As a result, even behaviorally consistent agents may explore less on some occasions, just because they were lucky and came across a good alternative early on. Such stochasticity can thus reduce the correlation between the same measure taken at two different time points, which would place an upper limit on the measure's capacity for test-retest reliability. To rule the possibility that test-retest reliability has a low upper bound by design, caused by stochasticity, we ran simulations and examined how the proposed measures of exploration in the bandit task (i.e., the switch rate and exploitation-complement rate) and the optional stopping task (i.e., number of boxes opened) are correlated across two different studies containing the same number of blocks as the study that we're proposing. Under the assumptions of the simulation models, the results provide an indication of the test-retest reliability of each measure. Note that we conducted these analyses for the tasks for which there were cognitive models available, and where it was feasible to model behavioural variability.

To this end, we simulated the behaviour of artificial agents acting repeatedly in a choice environment that closely mirrors our experimental settings (i.e., same number and parameterization of available options and same total number of trials). For the multi-armed bandit task we modeled artificial agents via an ε-greedy algorithm, commonly used for simulating behaviour in multi-armed bandit settings.[30] According to this algorithm, agents act under two possible, mutually exclusive modalities: at each trial, they either select the option that has yielded the largest payoff average (exploitation), or randomly select one of the options (random exploration). At each trial, the probability of making an exploiting action is indicated with ε (the unique free parameter of the model), and for the same agent it is constant. In the optional stopping task, we simulated agents behaving according to the optimal risk-averse solution of the problem, as in this task a single risk-aversion parameter controls the extent to which agents under or over-search.[31]

For the multi-armed bandit task, we simulated choice behaviour of a population of 1000 agents in two studies (thus for a total of 8 blocks of 40 trials each) with different values of ε (randomly drawn from the interval (0.25, 1). For each agent, we calculated the two behavioural measures of exploration for each block (i.e., the frequency of switches among options, and the complement to 1 of the frequency of selections of the choice with the largest average reward). For an indication

of test-retest reliability for each measure, we calculated the average exploration in the first four blocks (effectively reflecting a “Time 1” study) and the average in the last four blocks (reflecting a “Time 2” study), and correlated these with one another. For both measures, the test-retest reliability correlations were strong: $r = .95$ for the switch rate and $r = .96$ for the best-reply-complement (ps $< .001$).

For the optional stopping task, we simulated the behaviour of a population of 1000 agents in two studies with 8 search blocks each (4 conditions with costs 0.06,0.08,0.1,0.12, where each cost was repeated twice) and we looked at whether the number of boxes the agents opened in the two studies were correlated. This correlation reflects the test-retest reliability for the simulated agents. The agents had different risk-aversion parameters alpha, drawn uniformly from the interval (0.25,1.25). Values below 1 indicate risk-averse agents who search, on average, less than the optimal solution according to expected value theory (lower stopping threshold), and values above 1 indicate risk-seeking agents who search, on average, more than the optimal expected value solution (higher stopping threshold). The risk-aversion values we drew roughly correspond to the values observed in actual experiments on risk-aversion. That is, most people are risk averse or strongly risk averse, but there is a minority of people who are (mostly mildly) risk seeking. For this task we ran 1000 such simulations. The test-retest reliability correlations across the 1000 simulations ranged from $r = .58$ to $r = .69$ ($ps < 0.001$) with an average correlation of $r = .63$. This provides a rough estimate of the upper bound that we could expect to find in the proposed empirical study where there is stochasticity of the environment. We deem this upper bound quite sufficient.

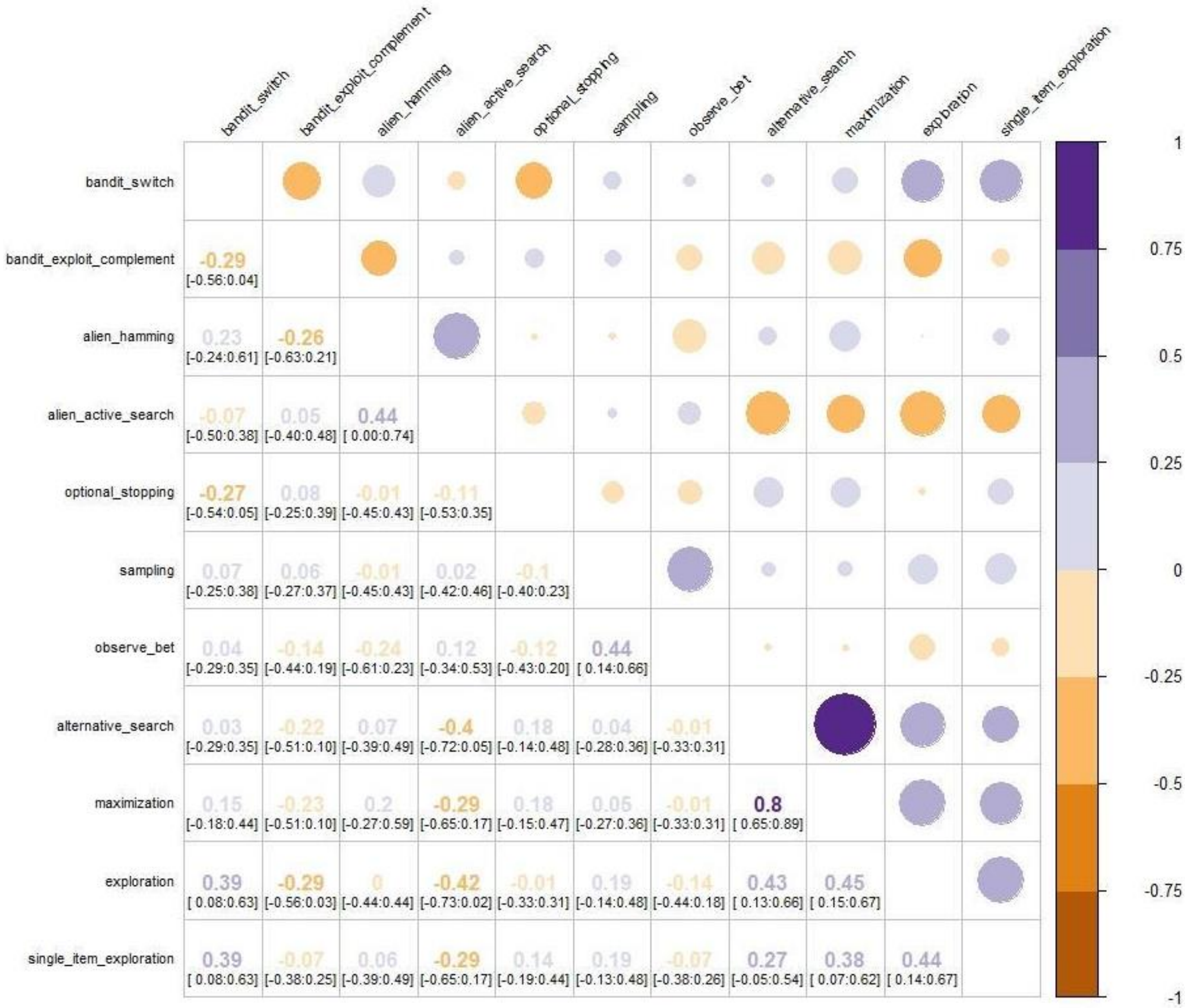

Figure S1. Pearson correlation coefficients from one-sided tests on the pilot data; uncorrected confidence intervals. Bottom-left are correlation coefficients (and 95% confidence intervals). Top-right are circles representing direction and size of the relationships (larger and more opaque means strong correlation). Purple numbers and circles reflect positive correlations and orange ones represent negative correlations.

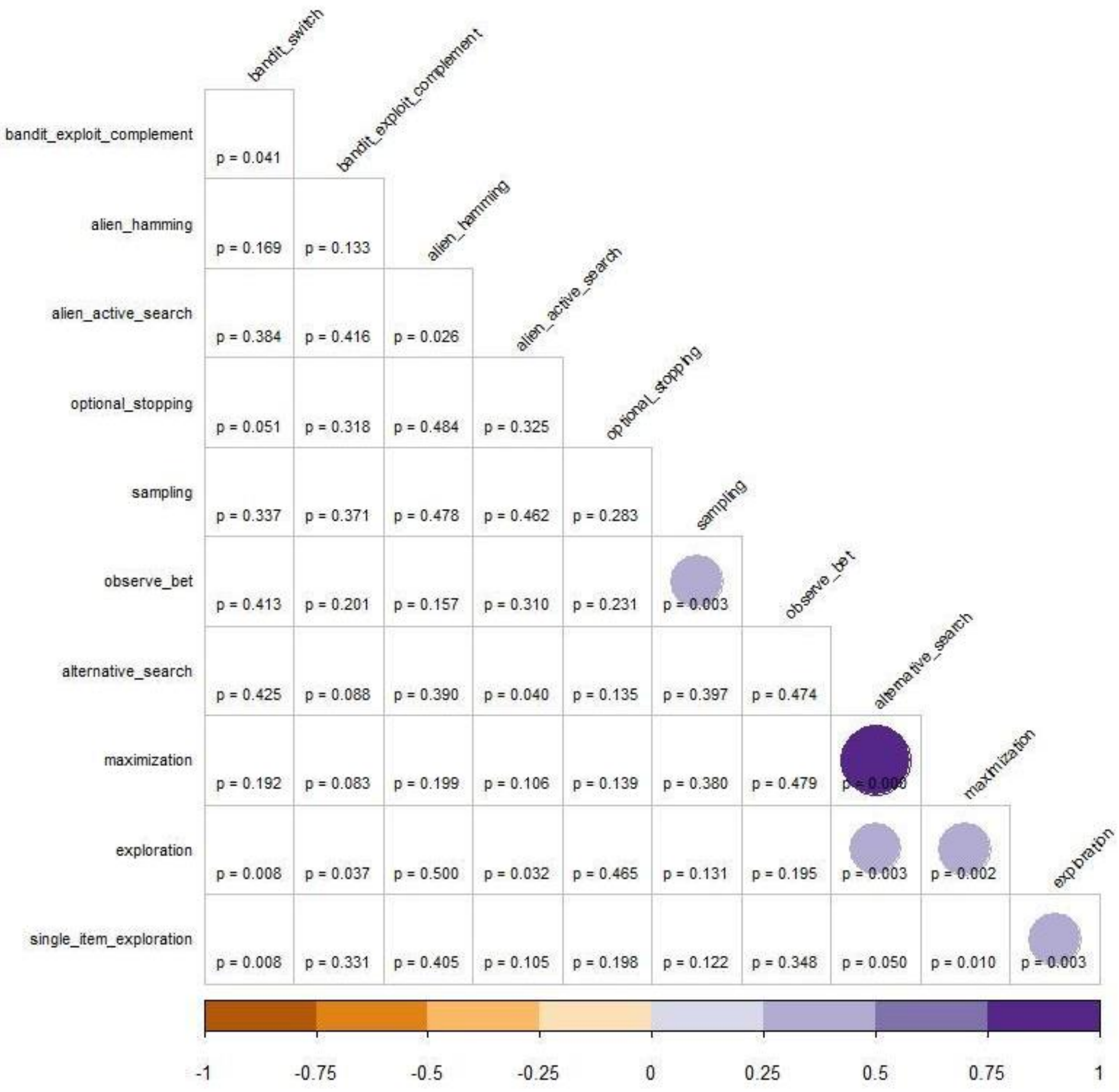

Figure S2. *P*-values from one-sided correlation tests for each pair of relationships; *p*-values were not adjusted for multiple comparisons; the corrected alpha for multiple comparisons for the pilot study was .005. Relationships that are significant at the corrected alpha (.005) are indicated by circles. The size and opaqueness of the circles indicates the strength and direction of the correlation. Purple circles reflect positive correlations and orange ones represent negative correlations.

Exploratory Self-Report Scales

For exploratory purposes, we included a general exploration question and the Big Five Inventory 2 (BFI-2)[32] as a measure of the Big Five personality traits.

The general exploration question asked participants, “Are you generally a person who makes the most of what you know and have or who explores new things and places, on a scale from 0 (“makes most of what I know/have”) to 10 (“explores new things”)?”. Answers range from 0-10 with higher scores reflecting a greater tendency to explore.

For the BFI-2, participants were given the following instructions followed by the personality items:

“Here are a number of characteristics that may or may not apply to you. For example, do you agree that you are someone who *likes to spend time with others*? Please write a number next to each statement to indicate the extent to which you agree or disagree with that statement.” (The items should be presented in the same order as they are numbered.)”

Each item was rated on a 5-point scale: 1=Disagree strongly, 2=Disagree a little, 3=Neutral; no opinion, 4=Agree a little, 5=Agree strongly. The BFI-2 consists of 60 items that fall into the five domains of personality, each of which consists of three facets. The items were presented in random order and are listed here grouped by the facet and domain for which they are averaged to produce a score (items with an r following the number are first reverse scored before averaging):

Extraversion
Sociability
1. Is outgoing, sociable.
46. Is talkative.
16r. Tends to be quiet.
31r. Is sometimes shy, introverted.
Assertiveness
6. Has an assertive personality.
21. Is dominant, acts as a leader.
36r. Finds it hard to influence people.
51r. Prefers to have others take charge.
Energy Level
41. Is full of energy.
56. Shows a lot of enthusiasm.
11r. Rarely feels excited or eager.
26r. Is less active than other people.

Agreeableness
Compassion
2. Is compassionate, has a soft heart.
32. Is helpful and unselfish with others.
17r. Feels little sympathy for others.
47r. Can be cold and uncaring.
Respectfulness
7. Is respectful, treats others with respect.
52. Is polite, courteous to others.
22r. Starts arguments with others.
37r. Is sometimes rude to others.

Trust
27. Has a forgiving nature.
57. Assumes the best about people.
12r. Tends to find fault with others.
42r. Is suspicious of others' intentions.

Conscientiousness
Organization
18. Is systematic, likes to keep things in order.
33. Keeps things neat and tidy.
3r. Tends to be disorganized.
48r. Leaves a mess, doesn't clean up.
Productiveness
38. Is efficient, gets things done.
53. Is persistent, works until the task is finished.
8r. Tends to be lazy.
23r. Has difficulty getting started on tasks.
Responsibility
13. Is dependable, steady.
43. Is reliable, can always be counted on.
28r. Can be somewhat careless.
58r. Sometimes behaves irresponsibly.

Negative Emotionality
Anxiety
19. Can be tense.
34. Worries a lot.
4r. Is relaxed, handles stress well.
49r. Rarely feels anxious or afraid.
Depression
39. Often feels sad.
54. Tends to feel depressed, blue.
9r. Stays optimistic after experiencing a setback.
24r. Feels secure, comfortable with self.
Emotional Volatility
14. Is moody, has up and down mood swings.
59. Is temperamental, gets emotional easily.
29r. Is emotionally stable, not easily upset.
44r. Keeps their emotions under control.

Open-Mindedness
Intellectual Curiosity
10. Is curious about many different things.
40. Is complex, a deep thinker.
25r. Avoids intellectual, philosophical discussions.
55r. Has little interest in abstract ideas.
Aesthetic Sensitivity
20. Is fascinated by art, music, or literature.
35. Values art and beauty.
5r. Has few artistic interests.
50r. Thinks poetry and plays are boring.

Creative Imagination
15. Is inventive, finds clever ways to do things.
60. Is original, comes up with new ideas.
30r. Has little creativity.
45r. Has difficulty imagining things.

Main Study: Robustness (Removing Extreme Responses)

We assessed the robustness of the results for Hypothesis 1 after removing participants who had an average score of 0 or 1 in either of the multi-armed bandit measures or a score of 0 or 10 in the active search measure from the alien game (i.e., the minimum and maximum scores on these measures). We didn’t remove extreme responses from the other measures because it is not unreasonable for the other measures to have extreme responses, whereas for the alien game, for example, a score of 0 for active search means the participant did not change any features across trials. Removing these participants made no substantive difference to the results in so far as the correlations were still mostly non-significant and did not show evidence for sufficient convergent validity for the behavioural measures. Figure S3 displays the results.

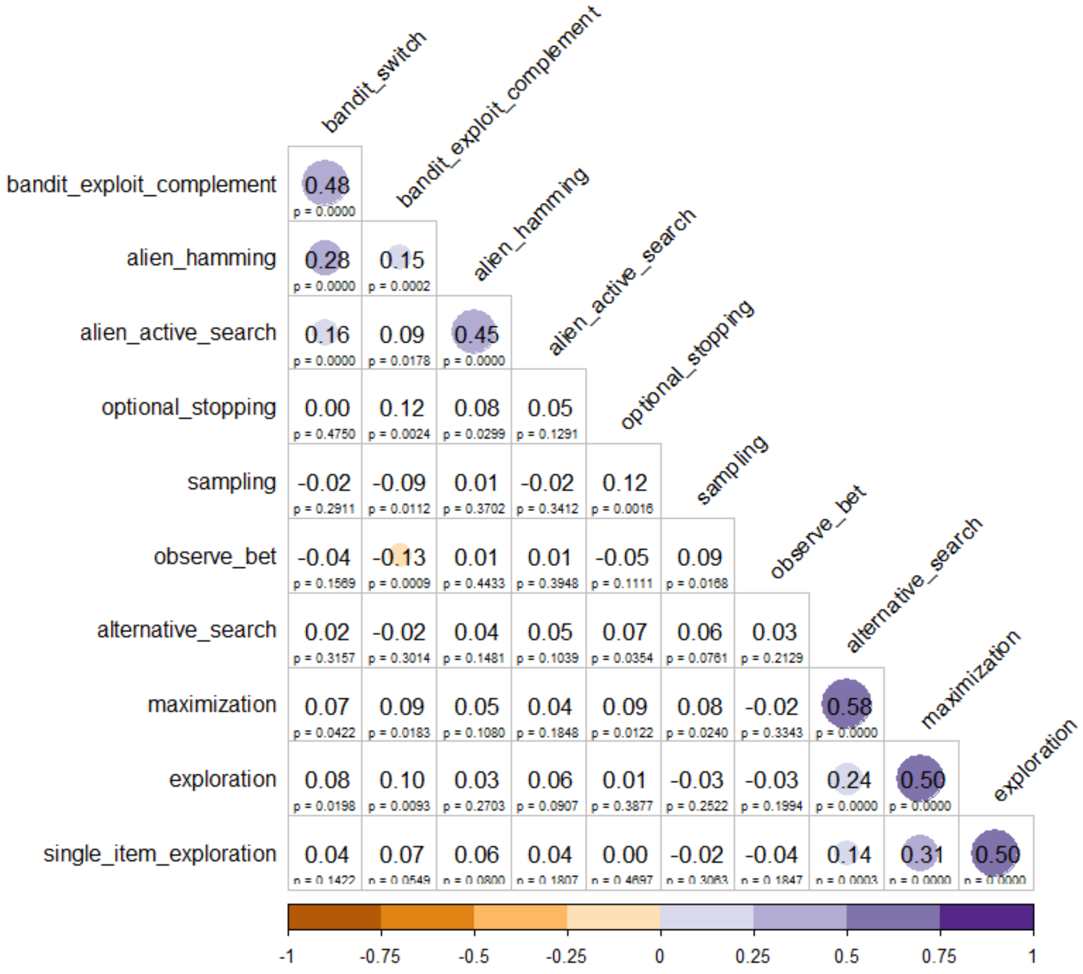

Figure S3. Pearson correlation coefficients (and one-sided *p* values) after removing extreme responses on multi-armed bandit and alien game measures; *p* values are not corrected for multiple comparisons; corrected alpha for multiple comparisons is .0009. The circles indicate that the correlation was statistically significant at the corrected alpha level of .0009 (larger and more opaque means stronger correlation).

Main Study: Check for Normality Assumption

We assessed multivariate normality with the tests of Mardia's skewness and kurtosis statistics, and univariate normality with the Shapiro-Wilk test. For multivariate normality, both p-values of skewness and kurtosis statistics were below 0.05, indicating that the data were not multivariate normal. For the Shapiro-Wilk tests, only the Maximization was not significant and, therefore, normally distributed. These conclusions do not change if we used Bonferroni corrected thresholds for the p-values. Taken together, the data were not multivariate normal and in the analyses of univariate normality only one variable had a normal distribution. Therefore, we used the WLSMV estimator to fit the factor models testing Hypothesis 2.

Main Study: Results with Exploratory Self-Report Scales

We conducted exploratory (one-sided correlation) analyses to test whether any of the domains of the personality questionnaire from the BFI-2 were correlated with any of the behavioural measures in the tasks. In these exploratory analyses, we included the age and gender variables (excluding the 5 participants who reported gender as other, to simplify the analyses and reporting). Figure S4 presents these results. Only one correlation between a BFI-2 domain and behavioural measure was statistically significant (at the corrected alpha level): Neuroticism was positively correlated with the number of trials in which participants chose to observe in the observe or bet task. People with higher ratings on Neuroticism were more likely to have a greater number of observe trials in that task (i.e., more exploratory). Age was negatively correlated with exploration in the observe or bet task: with increasing age, people tended to have fewer observe trials. None of the other correlations of the behavioural tasks with the BFI-2 domains, or with

age and gender, were statistically significant

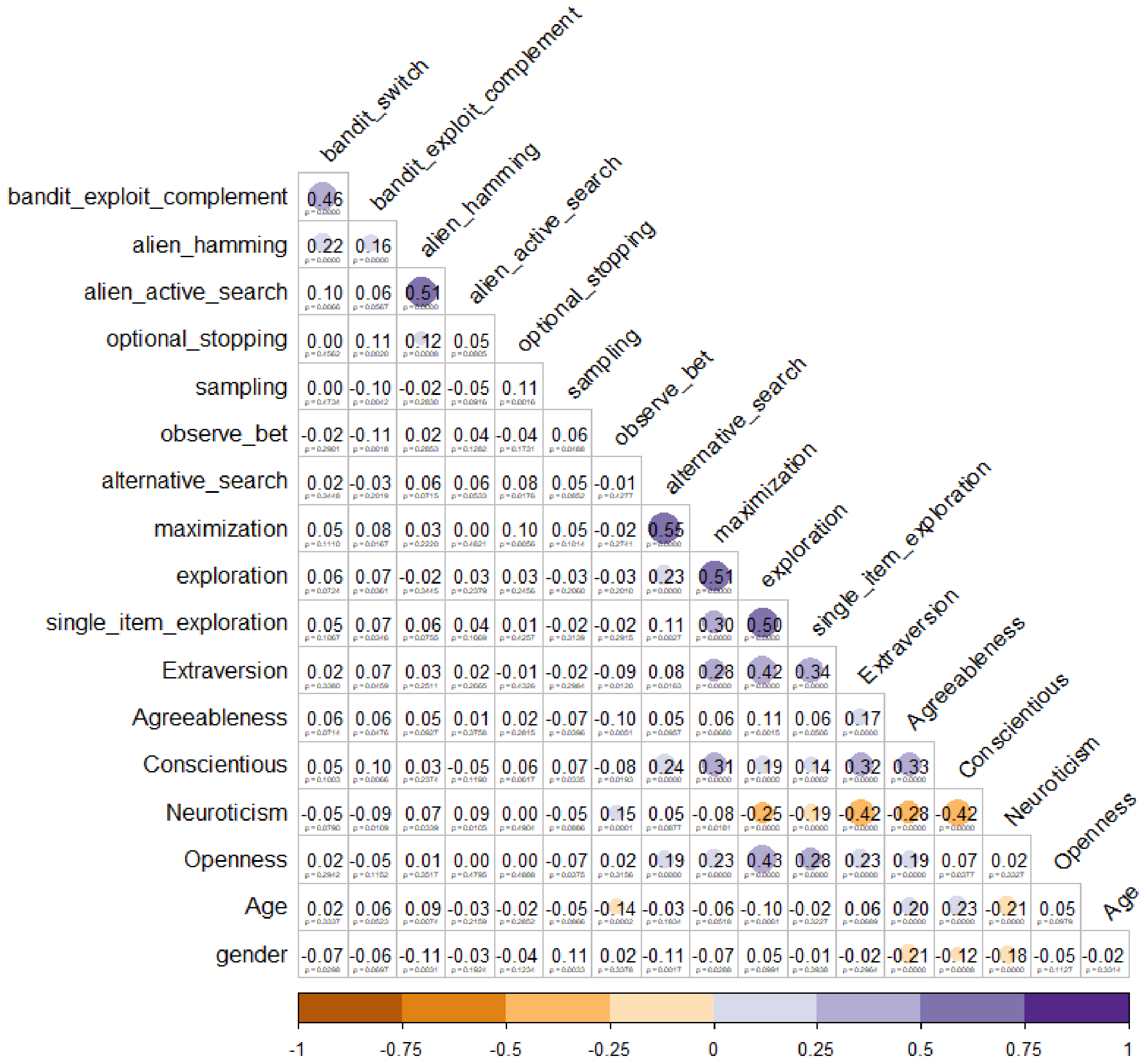

Figure S4. Pearson correlation coefficients (and one-sided *p* values) for all collected variables in the study; *p* values are not corrected for multiple comparisons; corrected alpha for multiple comparisons is .0009. The circles indicate that the correlation was statistically significant at the corrected alpha level of .0009 (larger and more opaque means stronger correlation).

Main Study: Non-Preregistered Exploratory Analyses for Hypothesis 2

Because the pre-registered models provided a poor fit to the aggregate scores and/or did not make sense from a theoretical perspective, we did non-preregistered analyses to identify and test another exploratory factor model. The model better reflects the correlation results from Hypothesis 1. We specified this model at the level of the blocks of each task and items of each self-report scale. That is, instead of using the aggregate scores of the tasks and scales, we developed a factor model that took into account each block (for the behavioural tasks) and item (for the self-report scales). Therefore, for each set of blocks that compromised a specific measure of exploration (e.g., the hamming distance at each block for the Alien task) we established a latent variable to represent that specific measure. The self-report measures, on the other hand, were modelled using a bifactor model. Because the self-report measures are highly correlated, we defined the group factors to represent method variance (i.e., bias due to the clustering of the items in a specific scale). The bifactor was used to represent the type of exploration-cognition that is measured by the self-report scales, including the general explore-exploit question. The complete model is represented in Figure S5.

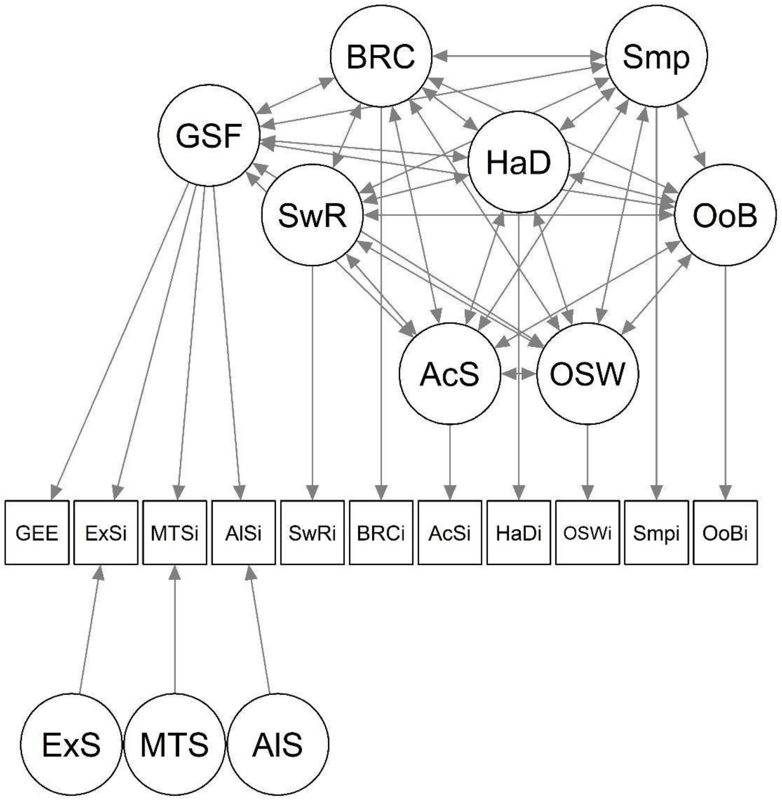

Figure S5. Representation of the block/item-level model. GSF: The general factor of the self-report scales. BRC: Bandit best-reply complement. SmP: sampling paradigm. SwR: Bandit switch rate. HaD: Hamming distance in alien game. OoB: Observe or bet. AcS: Active search in alien game. OSW: optional stopping with recall. ExS: Exploration Scale bias factor. MTS: Maximization Tendency Scale bias factor. AlS: Alternative Search Scale bias factor. GEE: general explore-exploit question. ExSi: items on the exploration scale. MTSi: items on the maximization tendency scale. AlSi: items on the alternative search scale. SwRi: switch-rate in the bandit task for each block separately. BRCi: best-reply complement in the bandit task for each block separately. AcSi: Active search in the alien game for each block separately. HaDi: Hamming distance in the alien game for each block separately. OSWi: number of boxes opened in optional stopping task for each block separately. Smpi: number of samples in sampling paradigm for each block separately. OoBi: number of observes in observe or bet task for each block separately.

We examined fit indices for this model using the training sample, the test sample, the test sample excluding those who did not participate at Time 2, and the Time 2 (T2) sample. Table S1

shows the fit indices. The model fit the data acceptably in every sample. Additionally, we fit a configural invariance model using (i) the test sample that excluded participants who did not complete the study at Time 2, and (ii) the Time 2 sample. This invariance model also showed acceptable fit.

Table S1

Fit indices for the non-preregistered exploratory model

Sample	χ^2	*df*	*p*	CFI	TLI	RMSEA	SRMR	ECVI
Training	1790.48	1485	< .0001	0.909	0.903	0.025	0.059	6.199
Test	1738.40	1485	< .0001	0.929	0.924	0.023	0.056	5.638
Test-Exclusions	1660.79	1485	.0009	0.935	0.931	0.022	0.061	6.963
Time 2	1723.03	1485	< .0001	0.903	0.896	0.025	0.062	7.018
Configural	3381.18	2970	< .0001	0.921	0.915	0.024	0.060	7.449

Note. Chi-square tests, CFI, TLI, RMSEA, SRMR, ECVI from Confirmatory Factor Analyses using the WLSMV estimator (because normality assumption did not hold) for the non-preregistered exploratory model (and configural model) in both the training and test samples, and in the Time 2 sample. Test- Exclusions = Test sample excluding participants who didn't participate at Time 2.

To test the invariance of this model we used the previously mentioned ΔCFI (threshold of -0.01), ΔGamma hat (threshold of -0.001), and ΔMcDonald's NCI (threshold of -0.02) indices. In Table S2 we see that the model can be considered (strictly) invariant according to ΔCFI and ΔGamma hat. But, the fit statistics slightly exceeded ΔMcDonald's NCI threshold suggesting that the model is not invariant. Therefore, we decided to use the bias-corrected bootstrap approach as preregistered for the main analyses. In this approach, one fits a configural model repeated times (in this case, 1,000) using random samples from the original sample, with equal group sizes to the original dataset. Then, one calculates the differences of the parameters, and bias correct the resulting sample of estimates. The last step is to calculate the 99% confidence intervals (CIs) generated with the bootstrap for each parameter. If the CI overlaps 0, then one concludes that the specific parameter is invariant. If the CI does not overlap 0, then that parameter is not invariant.

Table S2

Fit statistics for the invariance tests of the model

Model	CFI	ΔCFI	Gamma hat	ΔGamma hat	NCI	ΔNCI
Weak	0.932		0.976		1.118	
Strong	0.932	0.0003	0.976	0.0001	1.151	0.033
Strict	0.932	-0.0003	0.976	-0.0001	1.173	0.022

Note. Fit statistics (CFI, Gamma hat, NCI) for the measurement invariance tests of the non-preregistered exploratory model. ΔCFI = the difference in CFI between the model in this row and the model in the preceding row; ΔGamma hat = the difference in Gamma hat between the model in this row and the model in the preceding row; ΔNCI = the difference in NCI between the model in this row and the model in the preceding row.

The results of bias-corrected bootstrap approach are shown in Table S3. In this table, Lambda parameters are factor loadings, nu parameters are the intercepts, theta parameters are the uniqueness, and psi parameters are the latent correlations. Overall, we observe that most parameters are invariant. The only exceptions are the intercept of the first block of the switch-rate in the bandit task, the intercept of the first block of the exploit-complement in the bandit task, and the latent correlation between the Hamming distance in the alien game and samples in the sampling paradigm. Given that the majority of the parameters were invariant, we can conclude that the model is sufficiently invariant over time. Moreover, we believe this model to be more adequate to represent our data, as it does not force the behavioural tasks to be correlated and takes into account that the self-report tasks may have some important part of their variances explained by bias. In this sense, this model is more in line with what we found in the correlations tested between the aggregate scores of the tasks and scales.

Table S3

Bias Corrected Bootstrap 99% Confidence Intervals for assessing invariance

Parameter	lowerBound	Median	uppderBound	overlap0
Lambda_bandit_switch_1	-0.213	-0.025	0.144	TRUE
Lambda_bandit_switch_2	-0.094	0.022	0.142	TRUE
Lambda_bandit_switch_3	-0.089	0.041	0.203	TRUE
Lambda_bandit_switch_4	-0.125	0.037	0.183	TRUE
Lambda_bandit_exploit_complement_1	-0.165	-0.001	0.150	TRUE
Lambda_bandit_exploit_complement_2	-0.116	0.027	0.169	TRUE
Lambda_bandit_exploit_complement_3	-0.128	0.025	0.193	TRUE
Lambda_bandit_exploit_complement_4	-0.087	0.053	0.200	TRUE
Lambda_alien_hamming_1	-0.201	-0.027	0.157	TRUE
Lambda_alien_hamming_2	-0.219	-0.057	0.107	TRUE
Lambda_alien_hamming_3	-0.280	-0.057	0.146	TRUE
Lambda_alien_active_search_1	-0.126	0.037	0.175	TRUE
Lambda_alien_active_search_2	-0.126	0.023	0.158	TRUE
Lambda_alien_active_search_3	-0.137	0.038	0.221	TRUE
Lambda_optional_stopping_1	-0.262	0.075	0.460	TRUE
Lambda_optional_stopping_2	-0.428	-0.049	0.392	TRUE
Lambda_optional_stopping_3	-0.361	-0.045	0.391	TRUE
Lambda_optional_stopping_4	-0.333	0.019	0.409	TRUE
Lambda_optional_stopping_5	-0.764	-0.322	0.131	TRUE
Lambda_optional_stopping_6	-0.373	-0.027	0.369	TRUE
Lambda_optional_stopping_7	-0.559	-0.146	0.321	TRUE
Lambda_optional_stopping_8	-0.340	-0.023	0.332	TRUE
Lambda_sampling_1	-0.312	0.088	0.520	TRUE
Lambda_sampling_2	-0.217	0.070	0.327	TRUE
Lambda_sampling_3	-0.387	-0.029	0.244	TRUE
Lambda_sampling_4	-0.228	-0.035	0.165	TRUE
Lambda_sampling_5	-0.168	0.123	0.415	TRUE
Lambda_observe_bet_1	-0.561	-0.177	0.336	TRUE
Lambda_observe_bet_2	-0.408	0.085	0.559	TRUE
Lambda_alternativeSearch_1	-0.142	0.056	0.268	TRUE
Lambda_alternativeSearch_2	-0.168	0.052	0.275	TRUE

Lambda_alternativeSearch_3	-0.205	-0.025	0.166	TRUE
Lambda_alternativeSearch_4	-0.178	0.015	0.211	TRUE
Lambda_alternativeSearch_5	-0.146	0.021	0.184	TRUE
Lambda_alternativeSearch_6	-0.146	0.030	0.211	TRUE
Lambda_alternativeSearch_7	-0.193	-0.049	0.114	TRUE
Lambda_alternativeSearch_8	-0.270	-0.103	0.059	TRUE
Lambda_alternativeSearch_9	-0.086	0.072	0.245	TRUE
Lambda_alternativeSearch_10	-0.235	-0.050	0.155	TRUE
Lambda_alternativeSearch_11	-0.159	0.042	0.247	TRUE
Lambda_alternativeSearch_12	-0.092	0.106	0.301	TRUE
Lambda_maximizationTend_1	-0.252	0.048	0.334	TRUE
Lambda_maximizationTend_2	-0.285	0.039	0.347	TRUE
Lambda_maximizationTend_3	-0.276	0.026	0.348	TRUE
Lambda_maximizationTend_4	-0.388	-0.062	0.274	TRUE
Lambda_maximizationTend_5	-0.415	-0.069	0.259	TRUE
Lambda_maximizationTend_6	-0.721	-0.329	0.174	TRUE
Lambda_maximizationTend_7	-0.667	-0.052	0.500	TRUE
Lambda_maximizationTend_8	-0.692	-0.143	0.354	TRUE
Lambda_maximizationTend_9	-0.526	-0.151	0.286	TRUE
Lambda_explorationScale_1	-0.168	0.030	0.253	TRUE
Lambda_explorationScale_2	-0.038	0.123	0.274	TRUE
Lambda_explorationScale_3	-0.137	0.026	0.165	TRUE
Lambda_explorationScale_4	-0.112	0.026	0.174	TRUE
Lambda_explorationScale_5	-0.166	-0.011	0.116	TRUE
Lambda_alternativeSearch_1	-0.276	-0.069	0.151	TRUE
Lambda_alternativeSearch_2	-0.324	-0.106	0.108	TRUE
Lambda_alternativeSearch_3	-0.221	0.000	0.218	TRUE
Lambda_alternativeSearch_4	-0.233	-0.035	0.160	TRUE
Lambda_alternativeSearch_5	-0.203	0.004	0.201	TRUE
Lambda_alternativeSearch_6	-0.231	-0.039	0.137	TRUE
Lambda_alternativeSearch_7	-0.138	0.087	0.303	TRUE
Lambda_alternativeSearch_8	-0.173	0.033	0.257	TRUE
Lambda_alternativeSearch_9	-0.225	-0.038	0.138	TRUE
Lambda_alternativeSearch_10	-0.241	-0.025	0.195	TRUE
Lambda_alternativeSearch_11	-0.201	0.017	0.228	TRUE
Lambda_alternativeSearch_12	-0.299	-0.105	0.084	TRUE
Lambda_maximizationTend_1	-0.248	-0.050	0.135	TRUE
Lambda_maximizationTend_2	-0.196	-0.021	0.139	TRUE
Lambda_maximizationTend_3	-0.130	0.088	0.271	TRUE
Lambda_maximizationTend_4	-0.233	-0.037	0.126	TRUE
Lambda_maximizationTend_5	-0.230	-0.070	0.072	TRUE
Lambda_maximizationTend_6	-0.042	0.161	0.372	TRUE
Lambda_maximizationTend_7	-0.248	-0.014	0.242	TRUE
Lambda_maximizationTend_8	-0.147	0.034	0.236	TRUE
Lambda_maximizationTend_9	-0.017	0.179	0.377	TRUE
Lambda_explorationScale_1	-0.217	-0.039	0.124	TRUE
Lambda_explorationScale_2	-0.253	-0.015	0.220	TRUE
Lambda_explorationScale_3	-0.180	0.074	0.348	TRUE
Lambda_explorationScale_4	-0.261	0.007	0.270	TRUE
Lambda_explorationScale_5	-0.224	0.025	0.281	TRUE

Lambda_singleItemExp	-0.242	0.005	0.305	TRUE
Lambda_singleItemNearFarExp	-0.238	0.039	0.322	TRUE
**Nu_bandit_switch_1**	**0.052**	**0.273**	**0.495**	**FALSE**
Nu_bandit_switch_2	-0.154	0.083	0.282	TRUE
Nu_bandit_switch_3	-0.192	0.078	0.288	TRUE
Nu_bandit_switch_4	-0.170	0.054	0.254	TRUE
**Nu_bandit_exploit_complement_1**	**0.067**	**0.357**	**0.690**	**FALSE**
Nu_bandit_exploit_complement_2	-0.078	0.169	0.429	TRUE
Nu_bandit_exploit_complement_3	-0.060	0.197	0.438	TRUE
Nu_bandit_exploit_complement_4	-0.036	0.197	0.425	TRUE
Nu_alien_hamming_1	-0.132	0.042	0.209	TRUE
Nu_alien_hamming_2	-0.233	-0.016	0.163	TRUE
Nu_alien_hamming_3	-0.215	-0.017	0.178	TRUE
Nu_alien_active_search_1	-0.434	0.089	0.670	TRUE
Nu_alien_active_search_2	-0.567	-0.112	0.327	TRUE
Nu_alien_active_search_3	-0.271	0.161	0.657	TRUE
Nu_optional_stopping_1	-0.232	-0.037	0.158	TRUE
Nu_optional_stopping_2	-0.171	-0.009	0.155	TRUE
Nu_optional_stopping_3	-0.106	0.044	0.208	TRUE
Nu_optional_stopping_4	-0.169	0.005	0.192	TRUE
Nu_optional_stopping_5	-0.183	0.016	0.245	TRUE
Nu_optional_stopping_6	-0.238	-0.023	0.182	TRUE
Nu_optional_stopping_7	-0.106	0.082	0.284	TRUE
Nu_optional_stopping_8	-0.158	0.038	0.214	TRUE
Nu_sampling_1	-0.175	-0.058	0.056	TRUE
Nu_sampling_2	-0.144	-0.035	0.069	TRUE
Nu_sampling_3	-0.153	-0.037	0.079	TRUE
Nu_sampling_4	-0.175	-0.055	0.062	TRUE
Nu_sampling_5	-0.185	-0.067	0.064	TRUE
Nu_observe_bet_1	-0.221	-0.032	0.169	TRUE
Nu_observe_bet_2	-0.099	0.077	0.257	TRUE
Nu_alternativeSearch_1	-0.198	0.497	1.191	TRUE
Nu_alternativeSearch_2	-0.803	-0.178	0.393	TRUE
Nu_alternativeSearch_3	-0.823	-0.003	0.759	TRUE
Nu_alternativeSearch_4	-0.723	0.093	0.860	TRUE
Nu_alternativeSearch_5	-0.304	0.147	0.587	TRUE
Nu_alternativeSearch_6	-0.720	-0.063	0.523	TRUE
Nu_alternativeSearch_7	-0.756	-0.238	0.295	TRUE
Nu_alternativeSearch_8	-0.566	-0.200	0.214	TRUE
Nu_alternativeSearch_9	-0.699	-0.057	0.626	TRUE
Nu_alternativeSearch_10	-1.200	-0.183	0.727	TRUE
Nu_alternativeSearch_11	-0.695	0.180	1.011	TRUE
Nu_alternativeSearch_12	-1.250	-0.413	0.401	TRUE
Nu_maximizationTend_1	-1.268	-0.254	0.601	TRUE
Nu_maximizationTend_2	-0.721	-0.161	0.297	TRUE
Nu_maximizationTend_3	-0.380	0.159	0.675	TRUE
Nu_maximizationTend_4	-0.742	-0.129	0.463	TRUE
Nu_maximizationTend_5	-0.385	0.170	0.738	TRUE
Nu_maximizationTend_6	-0.496	-0.093	0.265	TRUE
Nu_maximizationTend_7	-0.279	0.489	1.215	TRUE

Nu_maximizationTend_8	-0.773	-0.110	0.608	TRUE
Nu_maximizationTend_9	-0.404	-0.020	0.340	TRUE
Nu_explorationScale_1	-1.584	-0.658	0.248	TRUE
Nu_explorationScale_2	-0.633	-0.146	0.350	TRUE
Nu_explorationScale_3	-0.916	-0.291	0.287	TRUE
Nu_explorationScale_4	-0.782	-0.359	0.033	TRUE
Nu_explorationScale_5	-0.512	-0.092	0.295	TRUE
Nu_singleItemExp	-0.770	-0.254	0.192	TRUE
Nu_singleItemNearFarExp	-0.478	-0.116	0.235	TRUE
Theta_bandit_switch_1	-0.224	0.041	0.310	TRUE
Theta_bandit_switch_2	-0.242	-0.040	0.167	TRUE
Theta_bandit_switch_3	-0.329	-0.071	0.157	TRUE
Theta_bandit_switch_4	-0.307	-0.064	0.204	TRUE
Theta_bandit_exploit_complement_1	-0.228	0.001	0.251	TRUE
Theta_bandit_exploit_complement_2	-0.262	-0.044	0.189	TRUE
Theta_bandit_exploit_complement_3	-0.290	-0.039	0.196	TRUE
Theta_bandit_exploit_complement_4	-0.304	-0.087	0.141	TRUE
Theta_alien_hamming_1	-0.242	0.045	0.320	TRUE
Theta_alien_hamming_2	-0.171	0.094	0.343	TRUE
Theta_alien_hamming_3	-0.243	0.092	0.445	TRUE
Theta_alien_active_search_1	-0.292	-0.062	0.218	TRUE
Theta_alien_active_search_2	-0.261	-0.038	0.203	TRUE
Theta_alien_active_search_3	-0.332	-0.058	0.195	TRUE
Theta_optional_stopping_1	-0.464	-0.077	0.287	TRUE
Theta_optional_stopping_2	-0.391	0.054	0.493	TRUE
Theta_optional_stopping_3	-0.400	0.053	0.409	TRUE
Theta_optional_stopping_4	-0.413	-0.020	0.345	TRUE
Theta_optional_stopping_5	-0.129	0.311	0.821	TRUE
Theta_optional_stopping_6	-0.472	0.034	0.467	TRUE
Theta_optional_stopping_7	-0.276	0.110	0.515	TRUE
Theta_optional_stopping_8	-0.306	0.023	0.355	TRUE
Theta_sampling_1	-0.627	-0.128	0.421	TRUE
Theta_sampling_2	-0.503	-0.113	0.331	TRUE
Theta_sampling_3	-0.383	0.049	0.525	TRUE
Theta_sampling_4	-0.272	0.059	0.378	TRUE
Theta_sampling_5	-0.601	-0.187	0.252	TRUE
Theta_observe_bet_1	-0.545	0.262	0.710	TRUE
Theta_observe_bet_2	-0.734	-0.119	0.544	TRUE
Theta_alternativeSearch_1	-0.163	0.019	0.196	TRUE
Theta_alternativeSearch_2	-0.122	0.044	0.215	TRUE
Theta_alternativeSearch_3	-0.106	0.036	0.163	TRUE
Theta_alternativeSearch_4	-0.125	0.012	0.164	TRUE
Theta_alternativeSearch_5	-0.193	-0.025	0.116	TRUE
Theta_alternativeSearch_6	-0.128	0.006	0.135	TRUE
Theta_alternativeSearch_7	-0.176	-0.019	0.132	TRUE
Theta_alternativeSearch_8	-0.062	0.119	0.263	TRUE
Theta_alternativeSearch_9	-0.177	-0.043	0.105	TRUE
Theta_alternativeSearch_10	-0.120	0.069	0.255	TRUE
Theta_alternativeSearch_11	-0.240	-0.055	0.138	TRUE
Theta_alternativeSearch_12	-0.174	-0.001	0.176	TRUE

Theta_maximizationTend_1	-0.157	0.046	0.248	TRUE
Theta_maximizationTend_2	-0.223	-0.007	0.201	TRUE
Theta_maximizationTend_3	-0.367	-0.116	0.136	TRUE
Theta_maximizationTend_4	-0.101	0.086	0.272	TRUE
Theta_maximizationTend_5	-0.117	0.096	0.324	TRUE
Theta_maximizationTend_6	-0.204	0.098	0.458	TRUE
Theta_maximizationTend_7	-0.720	-0.023	0.422	TRUE
Theta_maximizationTend_8	-0.668	-0.104	0.334	TRUE
Theta_maximizationTend_9	-0.338	-0.111	0.112	TRUE
Theta_explorationScale_1	-0.146	0.029	0.214	TRUE
Theta_explorationScale_2	-0.293	-0.146	0.023	TRUE
Theta_explorationScale_3	-0.209	-0.094	0.007	TRUE
Theta_explorationScale_4	-0.154	-0.044	0.069	TRUE
Theta_explorationScale_5	-0.102	0.006	0.105	TRUE
Theta_singleItemExp	-0.250	-0.005	0.207	TRUE
Theta_singleItemNearFarExp	-0.204	-0.027	0.143	TRUE
Psi_bandit_switch_bandit_exploit_complement	-0.156	0.037	0.210	TRUE
Psi_bandit_switch_alien_hamming	-0.253	0.020	0.279	TRUE
Psi_bandit_switch_alien_active_search	-0.442	-0.171	0.123	TRUE
Psi_bandit_switch_optional_stopping	-0.432	-0.037	0.325	TRUE
Psi_bandit_switch_sampling	-0.219	0.064	0.346	TRUE
Psi_bandit_switch_observe_bet	-0.300	-0.016	0.293	TRUE
Psi_bandit_switch_Self	-0.203	0.066	0.293	TRUE
Psi_bandit_exploit_complement_alien_hamming	-0.220	0.043	0.304	TRUE
Psi_bandit_exploit_complement_alien_active_search	-0.299	-0.055	0.226	TRUE
Psi_bandit_exploit_complement_optional_stopping	-0.134	0.144	0.460	TRUE
Psi_bandit_exploit_complement_sampling	-0.262	-0.003	0.238	TRUE
Psi_bandit_exploit_complement_observe_bet	-0.310	-0.034	0.235	TRUE
Psi_bandit_exploit_complement_Self	-0.261	-0.003	0.259	TRUE
Psi_alien_hamming_alien_active_search	-0.123	0.030	0.191	TRUE
Psi_alien_hamming_optional_stopping	-0.277	0.050	0.319	TRUE
**Psi_alien_hamming_sampling**	**-0.492**	**-0.259**	**-0.007**	**FALSE**
Psi_alien_hamming_observe_bet	-0.294	-0.004	0.286	TRUE
Psi_alien_hamming_Self	-0.092	0.159	0.384	TRUE
Psi_alien_active_search_optional_stopping	-0.239	0.085	0.360	TRUE
Psi_alien_active_search_sampling	-0.474	-0.180	0.074	TRUE
Psi_alien_active_search_observe_bet	-0.349	-0.088	0.176	TRUE
Psi_alien_active_search_Self	-0.020	0.225	0.485	TRUE
Psi_optional_stopping_sampling	-0.522	-0.181	0.131	TRUE
Psi_optional_stopping_observe_bet	-0.532	-0.226	0.057	TRUE
Psi_optional_stopping_Self	-0.316	0.018	0.372	TRUE
Psi_sampling_observe_bet	-0.406	-0.154	0.095	TRUE
Psi_sampling_Self	-0.117	0.127	0.379	TRUE
Psi_observe_bet_Self	-0.407	-0.116	0.124	TRUE

Note. Lambda parameters are factor loadings. Nu parameters are the intercepts. Theta parameters are the uniqueness. Psi parameters are the latent correlations. Confidence intervals that include zero are considered to be invariant. Confidence intervals with only negative values indicate that the parameter was larger in T2. Confidence intervals with only positive values indicate that the parameter was larger in T1.

Main Study: Exploratory Sensitivity Analysis of Differences Between Training and Testing Samples

The results shown in Table 4 of the main text indicate that the models provided a better fit for the testing sample. Because CFI, TLI, and RMSEA are sensitive to the values of the correlations between the variables, an improvement in one will be reflected by improvements also in the others. And it is quite likely that the random split, by pure chance, made the correlations in the testing sample somewhat larger than the correlations in the training sample. We did two analyses to assess the stability of our results. In short, our analyses showed that the differences in the indices between the testing and training samples occurred by chance (i.e., with 1,000 random splits we found that the fit statistics were in most cases marginally better in the training samples, and that confidence intervals for the fit statistics overlap).

The first analysis consisted in comparing the correlations of the measures with one another in the training sample against the same correlations in the test sample. We did this using *z* tests. This analysis is done by, first, transforming correlation coefficient values, or *r* values, into *z* scores with Fisher's transformation. Then, a *z* test statistic is calculated using

$$z = \frac{z_1 - z_2}{\sqrt{\frac{1}{N_1 - 3} + \frac{1}{N_2 - 3}}}$$

where z_1 is the z score of the training sample, z_2 is the z score of the testing sample, N_1 is the sample size of the training sample, and N_2 is the size of the testing sample. The results of this analysis are shown in Table S4.

It is possible to see that only four correlation differences were statistically significant when comparing the training and the testing sample at the threshold of .05 for the p-value. Three of these values were negative, indicating larger values in the testing sample. However, with a Bonferroni correction of the threshold (i.e., .05 / 55) for the p-value, no difference was significant. Therefore, it is reasonable to infer that the differences between fit in the training and testing samples observed in Table 4 are due to chance.

Table S4

Test statistics for the differences between the z scores in the training and testing samples

Row variable	Column variable	*r1*	*r2*	*N1*	*N2*	*z*	*p*	sig	Bonferroni
bandit_exploit_complement	bandit_switch	0.445	0.489	334	335	-0.718	0.473	FALSE	FALSE
alien_hamming	bandit_switch	0.272	0.192	332	331	1.075	0.283	FALSE	FALSE
alien_active_search	bandit_switch	0.152	0.043	332	331	1.416	0.157	FALSE	FALSE
optional_stopping	bandit_switch	-0.023	0.028	334	335	-0.652	0.515	FALSE	FALSE
sampling	bandit_switch	-0.103	0.080	329	330	-2.339	0.019	TRUE	FALSE
observe_bet	bandit_switch	-0.107	0.064	330	333	-2.198	0.028	TRUE	FALSE
alternative_search	bandit_switch	0.031	0.004	334	335	0.339	0.734	FALSE	FALSE

maximization	bandit_switch	0.063	0.047	334	335	0.210	0.834	FALSE	FALSE
exploration	bandit_switch	0.048	0.074	334	335	-0.344	0.731	FALSE	FALSE
single_item_exploration	bandit_switch	0.045	0.047	334	335	-0.020	0.984	FALSE	FALSE
alien_hamming	bandit_exploit_complement	0.154	0.177	332	331	-0.304	0.761	FALSE	FALSE
alien_active_search	bandit_exploit_complement	0.070	0.059	332	331	0.146	0.884	FALSE	FALSE
optional_stopping	bandit_exploit_complement	0.051	0.173	334	335	-1.595	0.111	FALSE	FALSE
sampling	bandit_exploit_complement	-0.175	-0.037	329	330	-1.792	0.073	FALSE	FALSE
observe_bet	bandit_exploit_complement	-0.192	-0.031	330	333	-2.089	0.037	TRUE	FALSE
alternative_search	bandit_exploit_complement	-0.059	-0.009	334	335	-0.650	0.516	FALSE	FALSE
maximization	bandit_exploit_complement	0.106	0.061	334	335	0.583	0.560	FALSE	FALSE
exploration	bandit_exploit_complement	0.104	0.037	334	335	0.870	0.384	FALSE	FALSE
single_item_exploration	bandit_exploit_complement	0.031	0.108	334	335	-0.998	0.318	FALSE	FALSE
alien_active_search	alien_hamming	0.545	0.487	337	335	1.021	0.307	FALSE	FALSE
optional_stopping	alien_hamming	0.103	0.130	337	335	-0.357	0.721	FALSE	FALSE
sampling	alien_hamming	0.063	-0.106	332	331	2.175	0.030	TRUE	FALSE
observe_bet	alien_hamming	0.043	-0.014	333	334	0.728	0.467	FALSE	FALSE
alternative_search	alien_hamming	0.043	0.059	337	335	-0.202	0.840	FALSE	FALSE
maximization	alien_hamming	0.019	0.057	337	335	-0.498	0.619	FALSE	FALSE
exploration	alien_hamming	-0.028	0.015	337	335	-0.562	0.574	FALSE	FALSE
single_item_exploration	alien_hamming	0.018	0.084	337	335	-0.858	0.391	FALSE	FALSE
optional_stopping	alien_active_search	0.067	0.040	337	335	0.355	0.723	FALSE	FALSE
sampling	alien_active_search	-0.019	-0.080	332	331	0.791	0.429	FALSE	FALSE
observe_bet	alien_active_search	0.104	-0.035	333	334	1.787	0.074	FALSE	FALSE
alternative_search	alien_active_search	0.043	0.075	337	335	-0.422	0.673	FALSE	FALSE
maximization	alien_active_search	-0.005	0.008	337	335	-0.168	0.867	FALSE	FALSE
exploration	alien_active_search	0.009	0.054	337	335	-0.581	0.561	FALSE	FALSE
single_item_exploration	alien_active_search	0.002	0.069	337	335	-0.861	0.389	FALSE	FALSE
sampling	optional_stopping	0.183	0.050	334	334	1.738	0.082	FALSE	FALSE
observe_bet	optional_stopping	-0.065	-0.007	335	337	-0.755	0.450	FALSE	FALSE
alternative_search	optional_stopping	0.064	0.097	339	339	-0.435	0.664	FALSE	FALSE
maximization	optional_stopping	0.089	0.102	339	339	-0.176	0.861	FALSE	FALSE
exploration	optional_stopping	0.013	0.038	339	339	-0.318	0.751	FALSE	FALSE
single_item_exploration	optional_stopping	-0.038	0.049	339	339	-1.124	0.261	FALSE	FALSE
observe_bet	sampling	0.067	0.056	330	333	0.140	0.888	FALSE	FALSE
alternative_search	sampling	0.055	0.049	334	334	0.083	0.934	FALSE	FALSE

maximization	sampling	0.043	0.069	334	334	-0.335	0.737	FALSE	FALSE
exploration	sampling	-0.063	0.008	334	334	-0.908	0.364	FALSE	FALSE
single_item_exploration	sampling	-0.056	0.023	334	334	-1.005	0.315	FALSE	FALSE
alternative_search	observe_bet	-0.020	0.012	335	337	-0.407	0.684	FALSE	FALSE
maximization	observe_bet	-0.031	-0.020	335	337	-0.140	0.888	FALSE	FALSE
exploration	observe_bet	-0.043	-0.028	335	337	-0.200	0.842	FALSE	FALSE
single_item_exploration	observe_bet	-0.046	0.010	335	337	-0.718	0.473	FALSE	FALSE
maximization	alternative_search	0.521	0.583	339	339	-1.163	0.245	FALSE	FALSE
exploration	alternative_search	0.233	0.225	339	339	0.108	0.914	FALSE	FALSE
single_item_exploration	alternative_search	0.036	0.180	339	339	-1.898	0.058	FALSE	FALSE
exploration	maximization	0.520	0.502	339	339	0.320	0.749	FALSE	FALSE
single_item_exploration	maximization	0.265	0.340	339	339	-1.062	0.288	FALSE	FALSE
single_item_exploration	exploration	0.463	0.540	339	339	-1.341	0.180	FALSE	FALSE

Note. Exploratory analyses. The table contains the results of two-sided *z* tests, comparing the correlations of the training sample (i.e., *r1*, with sample size *N1*) with the correlations of the testing sample (i.e., *r2*, with sample size *N2*) of the main study. The *p*-values are unadjusted for multiple comparisons; the Bonferroni corrected alpha is .0009.

The second analysis consisted in generating 1,000 random training and testing splits of the sample and calculating the mean and 95% confidence interval of the fit statistics of the models. The results of this analysis are shown in Table S5. Before discussing the results, we note that the correlations between the measures in the training sample are never going to be exactly the same as the correlations in the test sample. So it will always be the case that the correlations are either slightly higher or slightly lower in one sample than the other when the sample is split into two equal halves, assuming that the split into subsamples was random so that the relative correlations between the measures remains largely unchanged as compared to the correlations in the full sample (in this case, relative correlations being close to zero). Therefore, given the dependency of the fit statistics on the correlations between the measures, we would expect the fit statistics for all models to show a similar pattern when going from training to test samples. From Table S5, it is possible to see that, the fit statistics are very slightly better in the training sample than the test sample, but the confidence intervals almost always (10 out of 12 comparisons) overlap with the point estimates and all confidence intervals for all the fit statistics between the training and the testing samples overlap with one another. The fit statistics between the training and test samples can therefore not be statistically distinguished. Moreover, it is possible to see that the confidence intervals for all the fit statistics were better (i.e., larger CFI and TLI, and smaller RMSEA) for the Exploratory1 model, as compared to the Baseline3 model.

Table S5

Descriptive statistics of the 1,000 random splits of the sample

Model	Sample	Fit Statistic	Mean	SE	Lower Bound 95% CI	Upper Bound 95% CI
Baseline1	Training	CFI	0.4369	0.0020	0.4349	0.4389
	Testing		0.4367	0.0019	0.4348	0.4386
	Training	TLI	0.2961	0.0025	0.2936	0.2986
	Testing		0.2959	0.0024	0.2935	0.2983
	Training	RMSEA	0.1103	0.0002	0.1101	0.1105
	Testing		0.1106	0.0002	0.1104	0.1108
Baseline2	Training	CFI	0.7814	0.0013	0.7801	0.7828
	Testing		0.7806	0.0013	0.7793	0.7819
	Training	TLI	0.7205	0.0017	0.7188	0.7221
	Testing		0.7193	0.0017	0.7177	0.7210
	Training	RMSEA	0.0693	0.0002	0.0691	0.0695
	Testing		0.0695	0.0002	0.0693	0.0697
Baseline3	Training	CFI	0.9049	0.0014	0.9036	0.9063
	Testing		0.9040	0.0015	0.9026	0.9055
	Training	TLI	0.8416	0.0023	0.8393	0.8439
	Testing		0.8400	0.0024	0.8376	0.8425
	Training	RMSEA	0.0516	0.0004	0.0513	0.0520
	Testing		0.0519	0.0004	0.0515	0.0523
Exploratory1	Training	CFI	0.9537	0.0009	0.9528	0.9547
	Testing		0.9528	0.0009	0.9519	0.9537
	Training	TLI	0.9402	0.0013	0.9389	0.9414
	Testing		0.9389	0.0012	0.9376	0.9401
	Training	RMSEA	0.0302	0.0004	0.0298	0.0306
	Testing		0.0307	0.0004	0.0303	0.0311

Note. Exploratory analyses. SE: standard error. CI: confidence interval.

Main Study: Other Figures and Tables

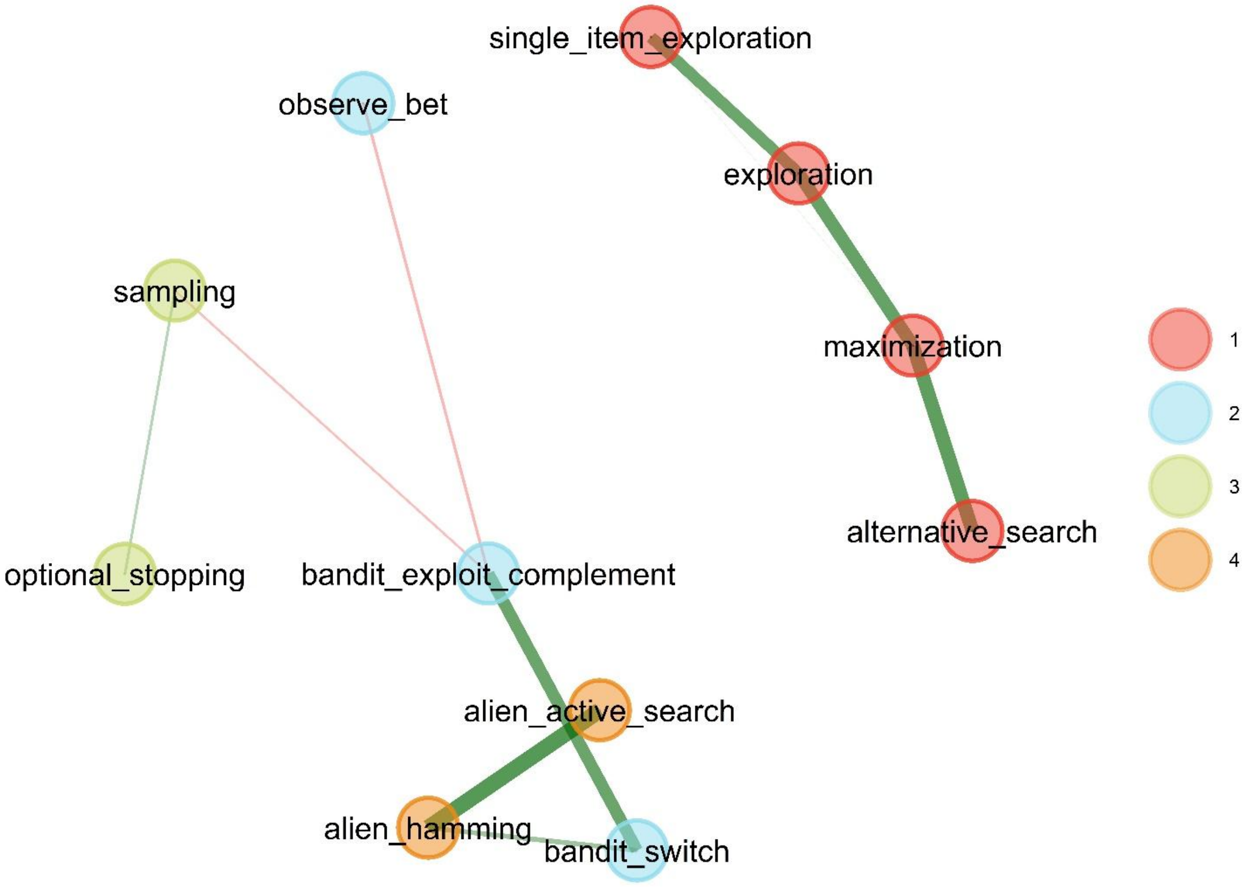

Figure S6. Exploratory model estimated with the Exploratory Graph Analysis (EGA) procedure. *Note*. Nodes of the same colour form a common factor. The edges connecting the nodes represent the regularised partial correlations. Green edges are positive partial correlations and red edges are negative partial correlations. The strength of the partial correlation is represented by the thickness of the edges, with thicker edges representing stronger partial correlations. These partial correlations are used within EGA to estimate the clusters of variables and are not used inferentially in any other means in this study.

Table S6

Summary of different behavioural tasks used to measure exploration

Task	Description	In this study
Multi-armed bandit	Participants choose between a given amount of options on each trial. Each option has a predefined payoff distribution. Participants see how many points they've won from the option they've chosen. How often participants change their choice across options from one trial to the next reflects how much they explore.	Yes

Alien game	Participants toggle on and off buttons (or decision attributes) that have to be configured jointly. Participants choose a combination of on and off buttons, and receive a reward associated with that button combination. How much they change the configuration of on and off buttons from one trial to the next reflects how much they explore.	Yes
Optional stopping with recall	Participants see a grid of options each hiding a predetermined stationary reward. Clicking on an option reveals the reward behind that option but comes at a cost. The costs accrue, so that revealing more options is more costly than revealing fewer options. Participants can choose when to stop revealing options, at which point they receive a reward equal to the highest paying option revealed so far minus the total accrued cost. The number of options a person reveals reflects how much they explore.	Yes
Sampling paradigm	Participants see two or more options each with some probability of paying out a reward. Participants can click the options without cost to learn about the options' payoff distribution. Once satisfied, participants choose which option they want to be paid out from. The number of times participants sample options to learn about their value reflects how much they explore.	Yes
Observe or bet	Participants see two lights on the screen and only one light turns on in any given trial with some predetermined probability. On each trial, participants can choose to observe which light turns on so that they can learn about the probabilities, or they can choose to bet whether one or the other light turns on. Observing means they see which light turns on but they earn no points. Betting means they don't see which light turns on but they get a point if they bet correctly and lose a point otherwise (they only know how many points they've won or lost at the end of the block of trials). The number of trials in which participants choose to observe reflects how much they explore.	Yes
Spatial foraging	Participants see a landscape with many options/locations on their screen and their search icon is placed in a starting location in the landscape. The landscape contains (deterministic) rewards at unknown locations. Participants must physically move the search icon through the landscape in search of the hidden rewards. The proportion of total surface area that is visited by participants reflects how much they explore (though there are also other measures of exploration and different types of foraging tasks.)	No
Secretary task	Participants are presented with a random sequence of options, one at a time, from a pool of options (the options are described as applicants for a secretary job). On each trial, participants choose to either reject the option they're presented with or to accept it. The pool of options is ranked from the worst to the best. The task is to accept the best option but participants can only accept the current option and can't go back to an option they've already rejected. The number of options participants search through reflects how much they explore.	No

Card search task	Participants are presented with a deck of cards face down (on the computer screen). Each card is associated with a predetermined value. Participants have a specified number of turns (e.g., 20) in which they can choose to either turn a new card over to see the points (i.e., explore) or to choose to earn the value of a card that they have already turned over (i.e., exploit). They can exploit the same card over multiple turns. The number of cards a participant turns over is the measure of how much they've explored.	No

Table S7

Descriptive statistics for each of the measures of exploration from the pilot data.

**Measure**	***M* (*SD*)**	***Range***
Bandit Switch	.59 (.22)	.07, .87
Bandit Exploit Complement	.57 (.26)	.14, 1.0
Alien Hamming	1.55 (1.07)	0.60, 3.90
Alien Active Search	6.18 (2.33)	1.00, 9.70
Optional Stopping	5.50 (2.86)	2.25, 17.63
Sampling Paradigm	12.47 (9.83)	2.20, 43
Observe or Bet	8.21 (7.07)	0, 36.5
Alternative Search Scale	4.38 (0.87)	2.3, 6
Maximization Tendency Scale	3.43 (0.75)	1.89, 5
Exploration Scale	3.70 (0.72)	2.2, 5
General Explore-Exploit Question	7 (1.87)	3, 10

Note. Bandit Switch = switch rate in multi-armed bandit task. Bandit Exploit Complement = the complement of the best reply rate in multi-armed bandit task. Alien Hamming = Hamming distance in alien game. Alien Active Search = active search measure in alien game. Optional Stopping = exploration in the optional stopping task. Sampling Paradigm = average number of samples per block in the sampling paradigm. Observe or Bet = average number of observe trials in the observe or bet task. Alternative Search Scale = mean ratings on the items of the alternative search scale. Maximization Tendency Scale = mean ratings on the items of the maximization tendency scale. Exploration Scale = mean ratings on the items of the exploration scale. General Explore-Exploit Question = ratings on the single-item measure of the general tendency to explore-exploit.

Table S8

Attributes of the Behavioural Tasks

	Multi-alternative	Distinct explore/exploit choices	Indirect exploration costs	Switching back and forth between explore and exploit	Completely resolving uncertainty about options after a choice	Multiple exploratio n measures	Multi-attribute	Rewards during exploration	Monetary explore-exploit trade-off	Computationally tractable problem solution
Multi-armed bandit	x		x	x		x		x	x	
Alien game	x	x	x	x	x	x	x	x	x	
Optional stopping	x	x			x				x	x
Sampling paradigm		x	x							
Observe-or-Bet		x	x	x					x	x

Note. ‘x’ indicates that the task has that structural quality.

Table S9

Factor loadings of the exploratory factor analysis with four retained factors, as suggested by a parallel analysis

Indicator	Fat1	Fat2	Fat3	Fat4
bandit_switch	0.005	0.184	**0.489**	0.010
bandit_exploit_complement	0.002	-0.017	**0.856**	-0.015
alien_hamming	0.008	**0.894**	0.020	-0.005
alien_active_search	-0.021	**0.619**	-0.040	0.038
optional_stopping	0.193	0.100	-0.006	-0.148
sampling	0.178	0.087	-0.221	-0.175
observe_bet	-0.029	0.130	-0.254	0.002
alternative_search	**0.647**	0.049	-0.077	-0.139
maximization	**0.866**	-0.019	0.038	0.089
exploration	0.235	-0.021	0.023	**0.621**
single_item_exploration	-0.089	0.032	-0.030	**0.692**

Table S10

Fit statistics for the invariance tests of the Exploratory1 model

Model	CFI	ΔCFI	Gamma hat	ΔGamma hat	NCI	ΔNCI
Configural	0.9713		0.9951		0.9951	
Weak	0.9740	0.0027	0.9955	0.0005	0.9958	0.0007
Strong	0.9766	0.0026	0.9960	0.0004	0.9987	0.0029
Strict	0.9728	-0.0038	0.9953	-0.0007	0.9957	-0.0030

Note. Fit statistics (CFI, Gamma hat, NCI) for the measurement invariance tests of the Exploratory1 model. ΔCFI = the difference in CFI between the model in this row and the model in the preceding row; ΔGamma hat = the difference in Gamma hat between the model in this row and the model in the preceding row; ΔNCI = the difference in NCI between the model in this row and the model in the preceding row.

Table S11

Question	Hypothesis	Sampling Plan<br>(e.g. power analysis)	Variables	Analysis Plan	Interpretation given to different outcomes	Outcome
Question 1 – Convergent Validity: Is there any convergent validity between different measures of exploratory behaviour?	Hypothesis 1-1: The switch-rate in the MAB will be positively correlated with the best-reply complement in the MAB.	With corrected alpha of 0.0009 and one-sided correlation tests, minimum required N = 556 to have 95% power to detect r = .2.<br><br>For the corresponding equivalence test, minimum required N = 632 to have 95% power to reject $\|r \geq .2\|$.<br><br>We will recruit 750 participants from Prolific.ac using their representative sample from the UK. We expect a final sample of 700 participants with complete data for all measures, after exclusions. However, if the number of participants is below 632, we will recruit another batch of participants so that the final sample for the analyses will be at least 632.	Switch-rate in MAB = the number of trials in which there's a switch from selecting one button to another button divided by the total number of trials in the block, averaged across the incentivized blocks.<br><br>Best-reply complement in MAB = the complement of one of the number of trials in which the option with the highest current average is selected divided by the total number of trials in the block, averaged across the incentivized blocks.	One-sided bivariate correlation tests against a null hypothesis of $r \leq 0$, with a corrected alpha of .0009.<br><br>Equivalence tests with the upper bound of r = .2 and lower bound of -1, with a corrected alpha of .0009. (Note, the equivalence tests will only be done if the correlation tests are statistically not significant.)<br><br>Cronbach's alpha will be used to assess internal consistency of each behavioural measure (using each block of each task as an observation) and self-report scale (using each item as an observation).	Statistically significant positive correlation = The switch-rate and best-reply complement have some convergent validity.<br>Statistically nonsignificant correlation = no evidence of convergent validity between the switch-rate and best-reply complement.<br>Statistically significant equivalence test = The switch-rate and best-reply complement do not have sufficient convergent validity.<br>Statistically nonsignificant equivalence test = cannot conclude that the switch-rate and best-reply complement are too weakly correlated for convergent validity.<br>Statistically significant negative correlation = The switch-rate and best-reply complement lack convergent validity with one another.	1-1: Hypothesis confirmed: switch-rated in MAB was positively correlated with the best-reply complement in MAB.<br>Question: The switch-rate and best-reply complement have some convergent validity.

		Participants who incorrectly answer any set of comprehension checks for a task more than 5 times (i.e., 6 incorrect attempts) will be excluded from analyses involving that task; only the first complete response of participants will be included.			Very low Cronbach’s alpha = the measure should be treated with caution as low internal consistency puts an upper bound on how strongly it can correlate with other measures.	

	Hypothesis 1-2: The switch-rate in the MAB will be positively correlated with the Hamming distance in the alien game.	Same as Hypothesis 1-1.	Switch-rate in MAB = the number of trials in which there's a switch from selecting one button to another button divided by the total number of trials in the block, averaged across the incentivized blocks.<br><br>Hamming distance in alien game = the number of attribute changes between the current combination and the best-performing prior combination, averaged across all trials and incentivized blocks.	Same as Hypothesis 1-1.	The same as Hypothesis 1-1 except with regards to the relationship between the switch-rate in MAB and the Hamming distance in alien game.	1-2: Hypothesis confirmed: switch-rated in MAB was positively correlated with Hamming distance in alien game. Question: The switch-rate and Hamming distance have some convergent validity.
	Hypothesis 1-3: The switch-rate in the MAB will be positively correlated with active search in the alien game.	Same as Hypothesis 1-1.	Switch-rate in MAB = the number of trials in which there's a switch from selecting one button to another button divided by the total number of trials in the block, averaged across the incentivized blocks.<br><br>Active search in alien game = the number of trials in a block in which the submitted configuration is different from the submitted configurations in all previous trials, averaged across incentivized blocks.	Same as Hypothesis 1-1.	The same as Hypothesis 1-1 except with regards to the relationship between the switch-rate in MAB and active search in alien game.	1-3: Hypothesis not confirmed: switch-rated in MAB was not significantly correlated with active search in alien game. The equivalence test was not statistically significant. Question: No evidence of convergent validity between the switch-rate and active search, and we cannot conclude that they have no convergent validity.

	Hypothesis 1-4: The switch-rate in the MAB will be positively correlated with the average number of boxes opened in the optional stopping task.	Same as Hypothesis 1-1.	Switch-rate in MAB = the number of trials in which there’s a switch from selecting one button to another button divided by the total number of trials in the block, averaged across the incentivized blocks.<br><br>Average number of boxes opened in optional stopping task: The number of boxes opened in each incentivized block of the optional stopping task divided by the total number of incentivized blocks.	Same as Hypothesis 1-1.	The same as Hypothesis 1-1 except with regards to the relationship between the switch-rate in MAB and boxes opened in the optional stopping task.	1-4: Hypothesis not confirmed: switch-rated in MAB was not significantly correlated with boxes opened in optional stopping task. The equivalence test was statistically significant.<br>Question: The switch-rate and number of boxes opened do not have sufficient convergent validity.
	Hypothesis 1-5: The switch-rate in the MAB will be positively correlated with samples in the sampling paradigm.	Same as Hypothesis 1-1.	Switch-rate in MAB = the number of trials in which there’s a switch from selecting one button to another button divided by the total number of trials in the block, averaged across the incentivized blocks.<br><br>Samples in sampling paradigm = number of samples in each incentivized block prior to final choice, divided by the total number of incentivized blocks.	Same as Hypothesis 1-1.	The same as Hypothesis 1-1 except with regards to the relationship between the switch-rate in MAB and number of samples in sampling paradigm.	1-5: Hypothesis not confirmed: switch-rated in MAB was not significantly correlated with number of samples in sampling paradigm. The equivalence test was statistically significant.<br>Question: The switch-rate and number of samples do not have sufficient convergent validity.
	Hypothesis 1-6: The switch-rate in the MAB will be positively correlated with observe trials in the observe or bet task.	Same as Hypothesis 1-1.	Switch-rate in MAB = the number of trials in which there’s a switch from selecting one button to another button divided by the total number of trials in the block, averaged across the incentivized blocks.<br><br>Observe trials = number of trials in each incentivized block of the observe or bet task where “observe” is chosen, divided	Same as Hypothesis 1-1.	The same as Hypothesis 1-1 except with regards to the relationship between the switch-rate in MAB and observe trials in the observe or bet task.	1-6: Hypothesis not confirmed: switch-rated in MAB was not significantly correlated with number of observe trials in observe or bet task. The equivalence test was statistically significant.<br>Question: The switch-rate and number of observe

			by the total number of incentivized blocks.			trials do not have sufficient convergent validity.
	Hypothesis 1-7: The switch-rate in the MAB will be positively correlated with mean self-report ratings on the exploration scale.	Same as Hypothesis 1-1.	Switch-rate in MAB = the number of trials in which there's a switch from selecting one button to another button divided by the total number of trials in the block, averaged across the incentivized blocks.<br><br>Mean self-report ratings on exploration scale.	Same as Hypothesis 1-1.	The same as Hypothesis 1-1 except with regards to the relationship between the switch-rate in MAB and self-report ratings on the exploration scale.	1-7: Hypothesis not confirmed: switch-rated in MAB was not significantly correlated with ratings on the exploration scale. The equivalence test was statistically significant.<br>Question: The switch-rate and the exploration scale do not have sufficient convergent validity.
	Hypothesis 1-8: The switch-rate in the MAB will be positively correlated with mean self-report ratings on the alternative search subscale.	Same as Hypothesis 1-1.	Switch-rate in MAB = the number of trials in which there's a switch from selecting one button to another button divided by the total number of trials in the block, averaged across the incentivized blocks.<br><br>Mean self-report ratings on alternative search subscale.	Same as Hypothesis 1-1.	The same as Hypothesis 1-1 except with regards to the relationship between the switch-rate in MAB and self-report ratings on the alternative search subscale.	1-8: Hypothesis not confirmed: switch-rated in MAB was not significantly correlated with ratings on the alternative search scale. The equivalence test was statistically significant.<br>Question: The switch-rate and the alternative search scale do not have sufficient convergent validity.

	Hypothesis 1-9: The switch-rate in the MAB will be positively correlated with mean self-report ratings on the maximization tendency scale.	Same as Hypothesis 1-1.	Switch-rate in MAB = the number of trials in which there's a switch from selecting one button to another button divided by the total number of trials in the block, averaged across the incentivized blocks.<br><br>Mean self-report ratings on maximization tendency scale.	Same as Hypothesis 1-1.	The same as Hypothesis 1-1 except with regards to the relationship between the switch-rate in MAB and self-report ratings on the maximization tendency scale.	1-9: Hypothesis not confirmed: switch-rated in MAB was not significantly correlated with ratings on the maximization tendency scale. The equivalence test was statistically significant.<br>Question: The switch-rate and the maximization tendency scale do not have sufficient convergent validity.
	Hypothesis 1-10: The switch-rate in the MAB will be positively correlated with self-report ratings on the general explore-exploit question.	Same as Hypothesis 1-1.	Switch-rate in MAB = the number of trials in which there's a switch from selecting one button to another button divided by the total number of trials in the block, averaged across the incentivized blocks.<br><br>Self-report ratings on the general explore-exploit question.	Same as Hypothesis 1-1.	The same as Hypothesis 1-1 except with regards to the relationship between the switch-rate in MAB and self-report ratings on the general explore-exploit question.	1-10: Hypothesis not confirmed: switch-rated in MAB was not significantly correlated with ratings on the general explore-exploit question. The equivalence test was statistically significant.<br>Question: The switch-rate and the general explore-exploit question do not have sufficient convergent validity.
	Hypothesis 1-11: The best-reply complement in the MAB will be positively correlated with the Hamming distance in the alien game.	Same as Hypothesis 1-1.	Best-reply complement in MAB.<br><br>Hamming distance in alien game.	Same as Hypothesis 1-1.	The same as Hypothesis 1-1 except with regards to the relationship between the best-reply complement in MAB and Hamming distance in alien game.	1-11: Hypothesis confirmed: best-reply complement in MAB was significantly correlated with the Hamming distance in alien game.<br>Question: The best-reply complement and the

						Hamming distance have some convergent validity.
	Hypothesis 1-12: The best-reply complement in the MAB will be positively correlated with active search in the alien game.	Same as Hypothesis 1-1.	Best-reply complement in MAB.<br><br>Active search in alien game.	Same as Hypothesis 1-1.	The same as Hypothesis 1-1 except with regards to the relationship between the best-reply complement in MAB and active search in alien game.	1-12: Hypothesis not confirmed: best-reply complement in MAB was not significantly correlated with active search in alien game. The equivalence test was statistically significant.<br>Question: The best-reply complement and active search do not have sufficient convergent validity.
	Hypothesis 1-13: The best-reply complement in the MAB will be positively correlated with average number boxes opened in optional stopping task.	Same as Hypothesis 1-1.	Best-reply complement in MAB.<br><br>Average number of boxes opened in optional stopping task.	Same as Hypothesis 1-1.	The same as Hypothesis 1-1 except with regards to the relationship between the best-reply complement in MAB and boxes opened in the optional stopping task.	1-13: Hypothesis not confirmed: best-reply complement in MAB was not significantly correlated with number of boxes opened in optional stopping task. The equivalence test was not statistically significant.<br>Question: No evidence of convergent validity between the best-reply complement and number of boxes opened and we

						cannot conclude that they lack convergent validity.
	Hypothesis 1-14: The best-reply complement in the MAB will be positively correlated with samples in sampling paradigm.	Same as Hypothesis 1-1.	Best-reply complement in MAB.<br><br>Number of samples in sampling paradigm.	Same as Hypothesis 1-1.	The same as Hypothesis 1-1 except with regards to the relationship between the best-reply complement in MAB and samples in sampling paradigm.	1-14: Hypothesis not confirmed: best-reply complement in MAB was not significantly correlated with number of samples in sampling paradigm. The equivalence test was statistically significant.<br>Question: The best-reply complement and number of samples do not have sufficient convergent validity.
	Hypothesis 1-15: The best-reply complement in the MAB will be positively correlated with observe trials in the observe or bet task.	Same as Hypothesis 1-1.	Best-reply complement in MAB.<br><br>Observe trials in observe or bet task.	Same as Hypothesis 1-1.	The same as Hypothesis 1-1 except with regards to the relationship between the best-reply complement in MAB and observe trials in observe or bet task.	1-15: Hypothesis not confirmed: best-reply complement in MAB was not significantly correlated with number of observe trials in observe or bet task. The equivalence test was statistically significant.<br>Question: The best-reply complement and number of observe trials do not have sufficient convergent validity.

	Hypothesis 1-16: The best-reply complement in the MAB will be positively correlated with mean self-report ratings on the exploration scale.	Same as Hypothesis 1-1.	Best-reply complement in MAB.<br><br>Mean self-report ratings on exploration scale.	Same as Hypothesis 1-1.	The same as Hypothesis 1-1 except with regards to the relationship between the best-reply complement in MAB and self-report ratings on the exploration scale.	1-16: Hypothesis not confirmed: best-reply complement in MAB was not significantly correlated with ratings on the exploration scale. The equivalence test was statistically significant. Question: The best-reply complement and exploration scale do not have sufficient convergent validity.
	Hypothesis 1-17: The best-reply complement in the MAB will be positively correlated with mean self-report ratings on the alternative search subscale.	Same as Hypothesis 1-1.	Best-reply complement in MAB.<br><br>Mean self-report ratings on the alternative search subscale.	Same as Hypothesis 1-1.	The same as Hypothesis 1-1 except with regards to the relationship between the best-reply complement in MAB and self-report ratings on the alternative search subscale.	1-17: Hypothesis not confirmed: best-reply complement in MAB was not significantly correlated with ratings on the alternative search scale. The equivalence test was statistically significant. Question: The best-reply complement and alternative search scale do not have sufficient convergent validity.
	Hypothesis 1-18: The best-reply complement in the MAB will be positively correlated with mean self-report ratings on the	Same as Hypothesis 1-1.	Best-reply complement in MAB.<br><br>Mean self-report ratings on maximization tendency scale.	Same as Hypothesis 1-1.	The same as Hypothesis 1-1 except with regards to the relationship between the best-reply complement in MAB and self-report ratings on the maximization tendency scale.	1-18: Hypothesis not confirmed: best-reply complement in MAB was not significantly correlated with ratings on the maximization tendency scale. The equivalence test was not statistically significant.

	maximization tendency scale.					Question: No evidence of convergent validity between the best-reply complement and the maximization tendency scale and we cannot conclude that they lack convergent validity.
	Hypothesis 1-19: The best-reply complement in the MAB will be positively correlated with self-report ratings on the general explore-exploit question.	Same as Hypothesis 1-1.	Best-reply complement in MAB.<br><br>Self-report ratings on the general explore-exploit question.	Same as Hypothesis 1-1.	The same as Hypothesis 1-1 except with regards to the relationship between the best-reply complement in MAB and self-report ratings on the general explore-exploit question.	1-19: Hypothesis not confirmed: best-reply complement in MAB was not significantly correlated with ratings on the genera explore-exploit question. The equivalence test was statistically significant.<br>Question: The best-reply complement and the general explore-exploit question do not have sufficient convergent validity.
	Hypothesis 1-20: The Hamming distance in the alien game will be positively correlated with active search in the alien game.	Same as Hypothesis 1-1.	Hamming distance in alien game.<br><br>Active search in alien game.	Same as Hypothesis 1-1.	The same as Hypothesis 1-1 except with regards to the relationship between the Hamming distance in alien game and active search in alien game.	1-20: Hypothesis confirmed: Hamming distance in alien game was significantly correlated with active search in the alien game.<br>Question: The Hamming distance and active search have convergent validity.

	Hypothesis 1-21: The Hamming distance in the alien game will be positively correlated with the average number of boxes opened in the optional stopping task.	Same as Hypothesis 1-1.	Hamming distance in alien game.<br><br>Average number of boxes opened in optional stopping task.	Same as Hypothesis 1-1.	The same as Hypothesis 1-1 except with regards to the relationship between the Hamming distance in alien game and boxes opened in optional stopping task.	1-21: Hypothesis not confirmed: Hamming distance in alien game was not significantly correlated with number of boxes opened in optional stopping task. The equivalence test was not statistically significant. Question: No evidence of convergent validity between the Hamming distance and number of boxes opened and we cannot conclude that they lack convergent validity.
	Hypothesis 1-22: The Hamming distance in the alien game will be positively correlated with samples in sampling paradigm.	Same as Hypothesis 1-1.	Hamming distance in alien game.<br><br>Samples in sampling paradigm.	Same as Hypothesis 1-1.	The same as Hypothesis 1-1 except with regards to the relationship between the Hamming distance in alien game and samples in sampling paradigm.	1-22: Hypothesis not confirmed: Hamming distance in alien game was not significantly correlated with number of samples in sampling paradigm. The equivalence test was statistically significant. Question: The Hamming distance and number of samples do not have sufficient convergent validity.

	Hypothesis 1-23: The Hamming distance in the alien game will be positively correlated with observe trials in the observe or bet task.	Same as Hypothesis 1-1.	Hamming distance in alien game.<br><br>observe trials in the observe or bet task.	Same as Hypothesis 1-1.	The same as Hypothesis 1-1 except with regards to the relationship between the Hamming distance in alien game and observe trials in the observe or bet task.	1-23: Hypothesis not confirmed: Hamming distance in alien game was not significantly correlated with observe trials in observe or bet task. The equivalence test was statistically significant.<br>Question: The Hamming distance and number of observe trials do not have sufficient convergent validity.
	Hypothesis 1-24: The Hamming distance in the alien game will be positively correlated with mean self-report ratings on the exploration scale.	Same as Hypothesis 1-1.	Hamming distance in alien game.<br><br>Mean self-report ratings on exploration scale.	Same as Hypothesis 1-1.	The same as Hypothesis 1-1 except with regards to the relationship between the Hamming distance in alien game and self-report ratings on the exploration scale.	1-24: Hypothesis not confirmed: Hamming distance in alien game was not significantly correlated with ratings on exploration scale. The equivalence test was statistically significant.<br>Question: The Hamming distance and exploration scale do not have sufficient convergent validity.
	Hypothesis 1-25: The Hamming distance in the alien game will be positively correlated with mean self-report ratings on the alternative search subscale.	Same as Hypothesis 1-1.	Hamming distance in alien game.<br><br>Mean self-report ratings on alternative search subscale.	Same as Hypothesis 1-1.	The same as Hypothesis 1-1 except with regards to the relationship between the Hamming distance in alien game and self-report ratings on the alternative search subscale.	1-25: Hypothesis not confirmed: Hamming distance in alien game was not significantly correlated with ratings on alternative search scale. The equivalence test was statistically significant.<br>Question: The Hamming distance and alternative

						search scale do not have sufficient convergent validity.
	Hypothesis 1-26: The Hamming distance in the alien game will be positively correlated with self-report ratings on the maximization tendency scale.	Same as Hypothesis 1-1.	Hamming distance in alien game.<br><br>Mean self-report ratings on the maximization tendency scale.	Same as Hypothesis 1-1.	The same as Hypothesis 1-1 except with regards to the relationship between the Hamming distance in alien game and self-report ratings on the maximization tendency scale.	1-26: Hypothesis not confirmed: Hamming distance in alien game was not significantly correlated with ratings on maximization tendency scale. The equivalence test was statistically significant.<br>Question: The Hamming distance and maximization tendency scale do not have sufficient convergent validity.
	Hypothesis 1-27: The Hamming distance in the alien game will be positively correlated with self-report ratings on the general explore-exploit question.	Same as Hypothesis 1-1.	Hamming distance in alien game.<br><br>Self-report ratings on the general explore-exploit question.	Same as Hypothesis 1-1.	The same as Hypothesis 1-1 except with regards to the relationship between the Hamming distance in alien game and self-report ratings on the general explore-exploit question.	1-27: Hypothesis not confirmed: Hamming distance in alien game was not significantly correlated with ratings on general explore-exploit question. The equivalence test was statistically significant.<br>Question: The Hamming distance and general explore-exploit question do not have sufficient convergent validity.

	Hypothesis 1-28: active search in the alien game will be positively correlated with the average number of boxes opened in the optional stopping task.	Same as Hypothesis 1-1.	Active search in alien game.<br><br>Average number of boxes opened in optional stopping task.	Same as Hypothesis 1-1.	The same as Hypothesis 1-1 except with regards to the relationship between the active search in alien game and boxes opened in the optional stopping task.	1-28: Hypothesis not confirmed: Active search in alien game was not significantly correlated with number of boxes opened in optional stopping task. The equivalence test was statistically significant. Question: Active search and number of boxes opened do not have sufficient convergent validity.
	Hypothesis 1-29: active search in the alien game will be positively correlated with samples in the sampling paradigm.	Same as Hypothesis 1-1.	Active search in alien game.<br><br>Samples in sampling paradigm.	Same as Hypothesis 1-1.	The same as Hypothesis 1-1 except with regards to the relationship between the active search in alien game and samples in sampling paradigm.	1-29: Hypothesis not confirmed: Active search in alien game was not significantly correlated with number of samples in sampling paradigm. The equivalence test was statistically significant. Question: Active search and number of samples do not have sufficient convergent validity.
	Hypothesis 1-30: active search in the alien game will be positively correlated with observe trials in the observe or bet task.	Same as Hypothesis 1-1.	Active search in alien game.<br><br>Observe trials in the observe or bet task.	Same as Hypothesis 1-1.	The same as Hypothesis 1-1 except with regards to the relationship between the active search in alien game and observe trials in the observe or bet task.	1-30: Hypothesis not confirmed: Active search in alien game was not significantly correlated with number of observe trials in observe or bet task. The equivalence test was statistically significant.

						Question: Active search and number of observe trials do not have sufficient convergent validity.
	Hypothesis 1-31: active search in the alien game will be positively correlated with mean self-report ratings on the exploration scale.	Same as Hypothesis 1-1.	Active search in alien game.<br><br>Mean self-report ratings on the exploration scale.	Same as Hypothesis 1-1.	The same as Hypothesis 1-1 except with regards to the relationship between the active search in alien game and self-report ratings on the exploration scale.	1-31: Hypothesis not confirmed: Active search in alien game was not significantly correlated with ratings on exploration scale. The equivalence test was statistically significant.<br>Question: Active search and exploration scale do not have sufficient convergent validity.
	Hypothesis 1-32: active search in the alien game will be positively correlated with mean self-report ratings on the alternative search subscale.	Same as Hypothesis 1-1.	Active search in alien game.<br><br>Mean self-report ratings on the alternative search subscale.	Same as Hypothesis 1-1.	The same as Hypothesis 1-1 except with regards to the relationship between the active search in alien game and self-report ratings on the alternative search subscale.	1-32: Hypothesis not confirmed: Active search in alien game was not significantly correlated with ratings on alternative search scale. The equivalence test was statistically significant.<br>Question: Active search and alternative search scale do not have sufficient convergent validity.

	Hypothesis 1-33: active search in the alien game will be positively correlated with mean self-report ratings on the maximization tendency scale.	Same as Hypothesis 1-1.	Active search in alien game.<br><br>Mean self-report ratings on the maximization tendency scale.	Same as Hypothesis 1-1.	The same as Hypothesis 1-1 except with regards to the relationship between the active search in alien game and self-report ratings on the maximization tendency scale.	1-33: Hypothesis not confirmed: Active search in alien game was not significantly correlated with ratings on maximization tendency scale. The equivalence test was statistically significant.<br>Question: Active search and maximization tendency scale do not have sufficient convergent validity.
	Hypothesis 1-34: active search in the alien game will be positively correlated with self-report ratings on the general explore-exploit question.	Same as Hypothesis 1-1.	Active search in alien game.<br><br>Self-report ratings on the general explore-exploit question.	Same as Hypothesis 1-1.	The same as Hypothesis 1-1 except with regards to the relationship between the active search in alien game and self-report ratings on the general explore-exploit question.	1-34: Hypothesis not confirmed: Active search in alien game was not significantly correlated with ratings on general explore-exploit question. The equivalence test was statistically significant.<br>Question: Active search and general explore-exploit question do not have sufficient convergent validity.
	Hypothesis 1-35: number of boxes opened in the optional stopping task will be positively correlated with samples in the	Same as Hypothesis 1-1.	Average number of boxes opened in optional stopping task.<br><br>Samples in sampling paradigm.	Same as Hypothesis 1-1.	The same as Hypothesis 1-1 except with regards to the relationship between the number of boxes opened in optional stopping task and number of samples in sampling paradigm.	1-35: Hypothesis not confirmed: Number of boxes opened in optional stopping task was not significantly correlated with number of samples in sampling paradigm. The

	sampling paradigm.					equivalence test was not statistically significant.<br>Question: No evidence of convergent validity between number of boxes opened and number of samples and we cannot conclude that they do not have convergent validity.
	Hypothesis 1-36: number of boxes opened in the optional stopping task will be positively correlated with observe trials in the observe or bet task.	Same as Hypothesis 1-1.	Average number of boxes opened in optional stopping task.<br><br>Observe trials in the observe or bet task.	Same as Hypothesis 1-1.	The same as Hypothesis 1-1 except with regards to the relationship between the number of boxes opened in optional stopping task and observe trials in the observe or bet task.	1-36: Hypothesis not confirmed: Number of boxes opened in optional stopping task was not significantly correlated with observe trials in observe or bet task. The equivalence test was statistically significant.<br>Question: Number of boxes opened and observe trials do not have sufficient convergent validity.
	Hypothesis 1-37: number of boxes opened in the optional stopping task will be positively correlated with mean self-report ratings on the exploration scale.	Same as Hypothesis 1-1.	Average number of boxes opened in optional stopping task.<br><br>Mean self-report ratings on exploration scale.	Same as Hypothesis 1-1.	The same as Hypothesis 1-1 except with regards to the relationship between the number of boxes opened in optional stopping task and self-report ratings on the exploration scale.	1-37: Hypothesis not confirmed: Number of boxes opened in optional stopping task was not significantly correlated with ratings on exploration scale. The equivalence test was statistically significant.<br>Question: Number of boxes opened and exploration scale do not

						have sufficient convergent validity.
	Hypothesis 1-38: number of boxes opened in the optional stopping task will be positively correlated with mean self-report ratings on the alternative search subscale.	Same as Hypothesis 1-1.	Average number of boxes opened in optional stopping task.<br><br>Mean self-report ratings on alternative search subscale.	Same as Hypothesis 1-1.	The same as Hypothesis 1-1 except with regards to the relationship between the number of boxes opened in optional stopping task and self-report ratings on the alternative search subscale.	1-38: Hypothesis not confirmed: Number of boxes opened in optional stopping task was not significantly correlated with ratings on alternative search scale. The equivalence test was statistically significant.<br>Question: Number of boxes opened and alternative search scale do not have sufficient convergent validity.
	Hypothesis 1-39: number of boxes opened in the optional stopping task will be positively correlated with mean self-report ratings on the maximization tendency scale.	Same as Hypothesis 1-1.	Average number of boxes opened in optional stopping task.<br><br>Mean self-report ratings on maximization tendency scale.	Same as Hypothesis 1-1.	The same as Hypothesis 1-1 except with regards to the relationship between the number of boxes opened in optional stopping task and self-report ratings on the maximization tendency scale.	1-39: Hypothesis not confirmed: Number of boxes opened in optional stopping task was not significantly correlated with ratings on maximization tendency scale. The equivalence test was not statistically significant.<br>Question: No evidence of convergent validity between number of boxes opened and maximization tendency scale and we cannot conclude that they do not have convergent validity.

	Hypothesis 1-40: number of boxes opened in the optional stopping task will be positively correlated with self-report ratings on the general explore-exploit question.	Same as Hypothesis 1-1.	Average number of boxes opened in optional stopping task.<br><br>Self-report ratings on the general explore-exploit question.	Same as Hypothesis 1-1.	The same as Hypothesis 1-1 except with regards to the relationship between the number of boxes opened in optional stopping task and self-report ratings on the general explore-exploit question.	1-40: Hypothesis not confirmed: Number of boxes opened in optional stopping task was not significantly correlated with ratings on the general explore-exploit question. The equivalence test was statistically significant. Question: Number of boxes opened and general explore-exploit question do not have sufficient convergent validity.
	Hypothesis 1-41: samples in sampling paradigm will be positively correlated with observe trials in the observe or bet task.	Same as Hypothesis 1-1.	Samples in sampling paradigm.<br><br>Observe trials in the observe or bet task.	Same as Hypothesis 1-1.	The same as Hypothesis 1-1 except with regards to the relationship between the number of samples in sampling paradigm and number of observe trials in observe or bet task.	1-41: Hypothesis not confirmed: Number of samples in sampling paradigm was not significantly correlated with observe trials in observe or bet task. The equivalence test was statistically significant. Question: Number of samples and number observe trials do not have sufficient convergent validity.

	Hypothesis 1-42: samples in sampling paradigm will be positively correlated with mean self-report ratings on the exploration scale.	Same as Hypothesis 1-1.	Samples in sampling paradigm.<br><br>Mean self-report ratings on the exploration scale.	Same as Hypothesis 1-1.	The same as Hypothesis 1-1 except with regards to the relationship between the number of samples in sampling paradigm and self-report ratings on the exploration scale.	1-42: Hypothesis not confirmed: Number of samples in sampling paradigm was not significantly correlated with ratings on the exploration scale. The equivalence test was statistically significant. Question: Number of samples and exploration scale do not have sufficient convergent validity.
	Hypothesis 1-43: samples in sampling paradigm will be positively correlated with mean self-report ratings on the alternative search subscale.	Same as Hypothesis 1-1.	Samples in sampling paradigm.<br><br>Mean self-report ratings on the alternative search subscale.	Same as Hypothesis 1-1.	The same as Hypothesis 1-1 except with regards to the relationship between the number of samples in sampling paradigm and self-report ratings on the alternative search subscale.	1-43: Hypothesis not confirmed: Number of samples in sampling paradigm was not significantly correlated with ratings on the alternative search scale. The equivalence test was statistically significant. Question: Number of samples and alternative search scale do not have sufficient convergent validity.
	Hypothesis 1-44: samples in sampling paradigm will be positively correlated with mean self-report ratings on the	Same as Hypothesis 1-1.	Samples in sampling paradigm.<br><br>Mean self-report ratings on the maximization tendency scale.	Same as Hypothesis 1-1.	The same as Hypothesis 1-1 except with regards to the relationship between the number of samples in sampling paradigm and self-report ratings on the maximization tendency scale.	1-44: Hypothesis not confirmed: Number of samples in sampling paradigm was not significantly correlated with ratings on the maximization tendency scale. The equivalence test

	maximization tendency scale.					was statistically significant. Question: Number of samples and maximization tendency scale do not have sufficient convergent validity.
	Hypothesis 1-45: samples in sampling paradigm will be positively correlated with self-report ratings on the general explore-exploit question.	Same as Hypothesis 1-1.	Samples in sampling paradigm.<br><br>Self-report ratings on the general explore-exploit question.	Same as Hypothesis 1-1.	The same as Hypothesis 1-1 except with regards to the relationship between the number of samples in sampling paradigm and self-report ratings on the general explore-exploit question.	1-45: Hypothesis not confirmed: Number of samples in sampling paradigm was not significantly correlated with ratings on the general explore-exploit question. The equivalence test was statistically significant. Question: Number of samples and general explore-exploit question do not have sufficient convergent validity.
	Hypothesis 1-46: observe trials in the observe or bet task will be positively correlated with mean self-report ratings on the exploration scale.	Same as Hypothesis 1-1.	Samples in sampling paradigm.<br><br>Mean self-report ratings on the exploration scale.	Same as Hypothesis 1-1.	The same as Hypothesis 1-1 except with regards to the relationship between the number of observe trials in observe or bet task and self-report ratings on the exploration scale.	1-46: Hypothesis not confirmed: Number of observe trials in observe or bet task was not significantly correlated with ratings on the exploration scale. The equivalence test was statistically significant. Question: Number of observe trials and exploration scale do not have sufficient convergent validity.

	Hypothesis 1-47: observe trials in the observe or bet task will be positively correlated with mean self-report ratings on the alternative search subscale.	Same as Hypothesis 1-1.	Samples in sampling paradigm.<br><br>Mean self-report ratings on the alternative search subscale.	Same as Hypothesis 1-1.	The same as Hypothesis 1-1 except with regards to the relationship between the number of observe trials in observe or bet task and self-report ratings on the alternative search subscale.	1-47: Hypothesis not confirmed: Number of observe trials in observe or bet task was not significantly correlated with ratings on the alternative search scale. The equivalence test was statistically significant.<br>Question: Number of observe trials and alternative search scale do not have sufficient convergent validity.
	Hypothesis 1-48: observe trials in the observe or bet task will be positively correlated with mean self-report ratings on the maximization tendency scale.	Same as Hypothesis 1-1.	Samples in sampling paradigm.<br><br>Mean self-report ratings on the maximization tendency scale.	Same as Hypothesis 1-1.	The same as Hypothesis 1-1 except with regards to the relationship between the number of observe trials in observe or bet task and self-report ratings on the maximization tendency scale.	1-48: Hypothesis not confirmed: Number of observe trials in observe or bet task was not significantly correlated with ratings on the maximization tendency scale. The equivalence test was statistically significant.<br>Question: Number of observe trials and maximization tendency scale do not have sufficient convergent validity.
	Hypothesis 1-49: observe trials in the observe or bet task will be positively correlated with	Same as Hypothesis 1-1.	Samples in sampling paradigm.<br><br>Self-report ratings on the general explore-exploit question.	Same as Hypothesis 1-1.	The same as Hypothesis 1-1 except with regards to the relationship between the number of observe trials in observe or bet task and self-report ratings on	1-49: Hypothesis not confirmed: Number of observe trials in observe or bet task was not significantly correlated with ratings on the general

	self-report ratings on the general explore-exploit question.				the general explore-exploit question.	explore-exploit question. The equivalence test was statistically significant.<br>Question: Number of observe trials and the general explore-exploit question do not have sufficient convergent validity.
	Hypothesis 1-50: mean self-report ratings on the exploration scale will be positively correlated with mean self-report ratings on the alternative search subscale.	Same as Hypothesis 1-1.	Mean self-report ratings on the exploration scale.<br><br>Mean self-report ratings on the alternative search subscale.	Same as Hypothesis 1-1.	The same as Hypothesis 1-1 except with regards to the relationship between self-report ratings on the exploration scale and self-report ratings on the alternative search subscale.	1-50: Hypothesis confirmed: ratings on the exploration scale were significantly correlated with ratings on the alternative search scale.<br>Question: The exploration scale and alternative search scale have some convergent validity.
	Hypothesis 1-51: mean self-report ratings on the exploration scale will be positively correlated with mean self-report ratings on the maximization tendency scale.	Same as Hypothesis 1-1.	Mean self-report ratings on the exploration scale.<br><br>Mean self-report ratings on the maximization tendency scale.	Same as Hypothesis 1-1.	The same as Hypothesis 1-1 except with regards to the relationship between self-report ratings on the exploration scale and self-report ratings on the maximization tendency scale.	1-51: Hypothesis confirmed: ratings on the exploration scale were significantly correlated with ratings on the maximization tendency scale.<br>Question: The exploration scale and maximization tendency scale have some convergent validity.
	Hypothesis 1-52: mean self-report ratings on the exploration scale will be positively	Same as Hypothesis 1-1.	Mean self-report ratings on the exploration scale.<br><br>Self-report ratings on the general explore-exploit question.	Same as Hypothesis 1-1.	The same as Hypothesis 1-1 except with regards to the relationship between self-report ratings on the exploration scale and self-	1-52: Hypothesis confirmed: ratings on the exploration scale were significantly correlated

	correlated with self-report ratings on the general explore-exploit question.				report ratings on the general explore-exploit question.	with ratings on the general explore-exploit question.<br>Question: The exploration scale and the general explore-exploit question have some convergent validity.
	Hypothesis 1-53: mean self-report ratings on the alternative search subscale will be positively correlated with mean self-report ratings on the maximization tendency scale.	Same as Hypothesis 1-1.	Mean self-report ratings on the alternative search subscale.<br><br>Mean self-report ratings on the maximization tendency scale.	Same as Hypothesis 1-1.	The same as Hypothesis 1-1 except with regards to the relationship between self-report ratings on the alternative search subscale and self-report ratings on the maximization tendency scale.	1-53: Hypothesis confirmed: ratings on the alternative search scale were significantly correlated with ratings on the maximization tendency scale.<br>Question: The alternative search scale and maximization tendency scale have some convergent validity.
	Hypothesis 1-54: mean self-report ratings on the alternative search subscale will be positively correlated with self-report ratings on the general explore-exploit question.	Same as Hypothesis 1-1.	Mean self-report ratings on the alternative search subscale.<br><br>Self-report ratings on the general explore-exploit question.	Same as Hypothesis 1-1.	The same as Hypothesis 1-1 except with regards to the relationship between self-report ratings on the alternative search subscale and self-report ratings on the general explore-exploit question.	1-54: Hypothesis not confirmed: ratings on the alternative search scale were not significantly correlated with ratings on the general explore-exploit question. The equivalence test was not statistically significant.<br>Question: No evidence of convergent validity between the alternative search scale and general explore-exploit question and we cannot conclude

						that they do not have convergent validity.
	Hypothesis 1-55: mean self-report ratings on the maximization tendency scale will be positively correlated with self-report ratings on the general explore-exploit question.	Same as Hypothesis 1-1.	Mean self-report ratings on the maximization tendency scale.<br><br>Self-report ratings on the general explore-exploit question.	Same as Hypothesis 1-1.	The same as Hypothesis 1-1 except with regards to the relationship between self-report ratings on the maximization tendency scale and self-report ratings on the general explore-exploit question.	1-55: Hypothesis confirmed: ratings on the maximization tendency scale were significantly correlated with ratings on the general explore-exploit question.<br>Question: The maximization tendency scale and general explore-exploit question have some convergent validity.
Question 2: Is there a domain-general tendency to explore that is captured by the behavioura l measures of exploration in the	Hypothesis 2: There will be evidence for a domain-general tendency to explore, *E*, to the extent that multiple behavioural measures (or a combination of behavioural and self-report measures) fit well	No power analysis was conducted for this hypothesis, but typically used rules of thumb (e.g., 10-15 observations per measure) are satisfied. The sampling plan is the same as for Question 1.<br><br>EFA: The exploratory models (see Analysis Plan column) will be derived using one half (approx. 350) of the observations	Switch-rate in MAB = the number of trials in which there's a switch from selecting one button to another button divided by the total number of trials in the block, averaged across the incentivized blocks.<br><br>Best-reply complement in MAB = the complement of one of the number of trials in which the option with the highest current average is selected divided by the total number of trials in the block, averaged across the incentivized blocks.	Our analyses for testing Hypothesis 2 includes three baseline confirmatory models and three exploratory data-driven models. For these analyses, we will randomly divide the data into two roughly equal parts (approx. N = 350 each). With one half of the data, we will first derive three	If the best fitting models in the CFA are those in which no two behavioural measures, or no combination of behavioural and self-report measures, fit well onto the same factor, or if we fail to identify any such models that fit the data well, then we will conclude that we did not find evidence for a latent variable that captures a domain general	2: The best fitting model in the CFA was one in which no two behavioural measures, and no combination of behavioural and self-report measures, fit well onto the same factor.<br>Question: We did not find evidence for a latent variable that captures a domain general behavioural tendency across tasks.

present study?	onto a common factor together.	randomly drawn from our final sample size of approximately 700.<br><br>CFA: The baseline and exploratory models (see Analysis Plan column) will be compared using confirmatory factor analysis in the remaining half (approx. 350) of the observations from our final sample size.<br><br>Participants who incorrectly answer any set of comprehension checks for a task more than 5 times will be excluded from all factor analyses (i.e., listwise deletion); only the first complete response of participants will be included.<br><br>As per the sampling plan for Hypotheses 1, we will aim for 700 participants with complete responses on all measures. Anticipating dropouts and exclusions, we will recruit 750 participants from Prolific.ac using their	Hamming distance in alien game = the number of attribute changes between the current combination and the best-performing prior combination, averaged across all trials and incentivized blocks.<br><br>Active search in alien game = the number of trials in a block in which the submitted configuration is different from the submitted configurations in all previous trials, averaged across incentivized blocks.<br><br>Average number of boxes opened in optional stopping task: The number of boxes opened in each incentivized block of the optional stopping task divided by the total number of incentivized blocks.<br><br>Samples in sampling paradigm = number of samples in each incentivized block prior to final choice, divided by the total number of incentivized blocks.<br><br>Observe trials = number of trials in each incentivized block of the observe or bet task where “observe” is chosen, divided by the total number of incentivized blocks.<br><br>Mean self-report ratings on exploration scale.	exploratory data-driven models and do a preliminary check of how well the confirmatory models fit the data. With the other half of the data, we will then compare all six models (the three baseline confirmatory models and the three data-driven models) against each other with confirmatory factor analysis (CFA).<br><br>Baseline1: The first baseline model assumes a general factor representing the tendency to explore, thus excluding any methodological effect of the elicitation methods of explorative behavior.<br><br>Baseline2: the explained variance is accounted for by only two specific oblique factors reflecting the methodologies of measurement (i.e.,	behavioural tendency across tasks. There will be evidence for a domain-general tendency to explore, *E*, to the extent that multiple behavioural measures (or a combination of behavioural and self-report measures) fit well onto a common factor together. The strongest evidence for *E*, and the validity of the measures in capturing it, will be if a single factor can be extracted that explains variance in all of the 11 measures of exploration.	

		representative sample from the UK.	Mean self-report ratings on alternative search subscale.<br><br>Mean self-report ratings on maximization tendency scale.<br><br>Self-report ratings on the general explore-exploit question.	behavioral and self-report measures).<br><br>Baseline3: the explained variance is accounted for by two orthogonal factors reflecting common method variance for the behavioral tasks and the self-report measures, and one common underlying factor representing the tendency to explore.<br><br>Exploratory1: the best model selected based on exploratory graph analysis and a combination of parallel analysis and exploratory factor analysis (with minimum absolute loading of a measure on a factor being 0.30). Model selection is based on parsimonious theoretical justification.<br><br>Exploratory2: a bifactor model with a general tendency to explore that loads to all		

				measures but that also includes the factors identified in Exploratory1 loading to their respective measures.<br><br>Exploratory 3: a bifactor model derived from exploratory bifactor analysis with the model selected from a parsimonious theoretical perspective.<br><br>***Confirmatory factor analysis: Model comparisons***.<br>We will then use the remaining random half of our data to fit and compare, using CFA, Baseline1, Baseline2, Baseline3, Exploratory1, Exploratory2, and Exploratory3. For the EFA-derived models (i.e., Exploratory1-3), absolute loadings below 0.30 will be fixed as 0. If the data from the tasks are normally distributed, parameter estimation		

				will be done with robust Maximum Likelihood estimation (MLR). If the normality assumption does not hold, parameter estimation will be done with the Weighted Least Square Mean and Variance Adjusted Estimators.<br><br>The models will be compared with regard to fit indices commonly used in the context of model comparison in factor analysis: CFI; TLI; and RMSEA. The best fitting model will therefore be the one with CFI and TLI closest to 1 and RMSEA closest to 0. If two or more models provide a very similar fit to the data, the model we select will be the one that makes the most sense from a parsimonious theoretical perspective.		

Question 3: Do the behavioural measures of exploration in the present study capture a trait-like construct that is stable across time?	Hypothesis 3-0: the measurement model derived from addressing Question 2 will be invariant across time.	Same as Hypothesis 3-1.	The measures retained in the measurement model selected from the analyses for answering Question 2.	We will examine the differences between fit indices of the configural, weak factorial, strong factorial, and strict variance models. The fit indices we will use are ΔCFI, ΔGamma hat, and ΔMcDonald's NCI. As decision criteria, the thresholds are a ΔCFI larger than –0.01, a ΔGamma hat larger than –.001, and ΔMcDonald's NCI larger than –.02. If at any level we cannot establish invariance for the entire construct, we will use the bias-corrected bootstrap confidence intervals approach to identify and remove any problematic tasks and try to establish partial measurement invariance for the remaining tasks at the same level. The bias-corrected bootstrap confidence intervals approach to measurement	Fit indices that cross the thresholds indicate that the null hypothesis of invariance should be rejected and that the measurement model is thus not invariant across time. If it is required to use the bias-corrected bootstrap confidence intervals, and a confidence interval does not include zero it can be inferred that the specific parameter is not invariant.	3-0: Hypothesis was confirmed: The measurement model retained from answering Question 2 was invariance across time.<br>Question: The measurement model identified in Question was stable across time.

				invariance is centred on evaluating the confidence intervals for all the differences between parameters in the model.<br><br>The analyses will be run only with participants who complete the measures at both Time 1 and Time 2.		
	Hypothesis 3-1: the switch-rate in the MAB at Time 1 will be positively correlated with the switch-rate in the MAB at Time 2.	After first participation (Time 1), we will invite the participants whose data we use for the confirmatory factor analyses of Hypothesis 2 (approx. N = 350) to take part in the same study one month later (Time 2). We expect Time 2 to result in around 300 complete responses. This will give the one-sided correlation tests 95% power to detect a positive correlation of r = .188.<br><br>Participants who incorrectly answer any set of comprehension checks	Switch-rate in MAB at Time 1 = the number of trials in which there's a switch from selecting one button to another button divided by the total number of trials in the block, averaged across the incentivized blocks at Time 1.<br><br>Switch-rate in MAB at Time 2 = the number of trials in which there's a switch from selecting one button to another button divided by the total number of trials in the block, averaged across the incentivized blocks at Time 2.	We will run a one-sided pairwise bivariate correlation test against a null hypothesis of $r \leq 0$, with an alpha of .05.<br><br>The analyses will be run only with participants who complete the measures at both Time 1 and Time 2.	Statistically significant positive correlation: the switch-rate in the MAB has some test-retest reliability.<br><br>Statistically nonsignificant correlation: we have no evidence that the switch-rate in the MAB has test-retest reliability.<br><br>Note that there is no threshold for reliability; we therefore do not have a smallest effect size of interest to use for power analysis or equivalence testing. Nonetheless, for each measure, the strength	3-1: Hypothesis was confirmed: Switch-rate in MAB at Time 1 was positively correlated with switch-rate at Time 2. Question: The switch-rate has test-retest reliability.

		for a task more than 5 times will be excluded from analyses involving that task; only the first complete response of participants will be included.			of the correlation gives a measure of its reliability.	
	Hypothesis 3-2: the best-reply complement in the MAB at Time 1 will be positively correlated with the best-reply complement in the MAB at Time 2.	Same as Hypothesis 3-1.	Best-reply complement in MAB at Time 1 = the complement of one of the number of trials in which the option with the highest current average is selected divided by the total number of trials in the block, averaged across the incentivized blocks at time 1.<br><br>Best-reply complement in MAB at Time 2 = the complement of one of the number of trials in which the option with the highest current average is selected divided by the total number of trials in the block, averaged across the incentivized blocks at Time 2.	Same as Hypothesis 3-1	Same as Hypothesis 3-1 but with regards to the best-reply complement in the MAB.	3-2: Hypothesis was confirmed: Best-reply complement at Time 1 was positively correlated with best-reply complement at Time 2.<br>Question: The best-reply complement has test-retest reliability.
	Hypothesis 3-3: the Hamming distance in the alien game at Time 1 will be positively correlated with the Hamming distance in the	Same as Hypothesis 3-1.	Hamming distance in alien game at Time 1 = the number of attribute changes between the current combination and the best-performing prior combination, averaged across all trials and incentivized blocks at time 1.<br><br>Hamming distance in alien game at Time 2 = the number of attribute changes between the current combination and the	Same as Hypothesis 3-1	Same as Hypothesis 3-1 but with regards to the Hamming distance in the alien game.	3-3: Hypothesis was confirmed: Hamming distance at Time 1 was positively correlated with Hamming distance at Time 2.<br>Question: The Hamming distance has test-retest reliability.

	alien game at Time 2.		best-performing prior combination, averaged across all trials and incentivized blocks at time 2.			
	Hypothesis 3-4: active search in the alien game at Time 1 will be positively correlated with active search in the alien game at Time 2.	Same as Hypothesis 3-1.	Active search in alien game at Time 1 = the number of trials in a block in which the submitted configuration is different from the submitted configurations in all previous trials, averaged across incentivized blocks at time 1.<br><br>Active search in alien game at Time 2 = the number of trials in a block in which the submitted configuration is different from the submitted configurations in all previous trials, averaged across incentivized blocks at time 2.	Same as Hypothesis 3-1	Same as Hypothesis 3-1 but with regards to active search in the alien game.	3-4: Hypothesis was confirmed: Active search at Time 1 was positively correlated with Active search at Time 2. Question: Active search has test-retest reliability.
	Hypothesis 3-5: number of boxes opened in the optional stopping task at Time 1 will be positively correlated with number of boxes opened in the optional stopping task at Time 2.	Same as Hypothesis 3-1.	Average number of boxes opened in optional stopping task at Time 1 = the number of boxes opened in each incentivized block of the optional stopping task divided by the total number of incentivized blocks at time 1.<br><br>Average number of boxes opened in optional stopping task at Time 2 = the number of boxes opened in each incentivized block of the optional	Same as Hypothesis 3-1	Same as Hypothesis 3-1 but with regards to number of boxes opened in the optional stopping task.	3-5: Hypothesis was confirmed: Number of boxes opened in optional stopping task at Time 1 was positively correlated with number of boxes opened at Time 2. Question: Number of boxes opened has test-retest reliability.

			stopping task divided by the total number of incentivized blocks at time 2.			
	Hypothesis 3-6: samples in the sampling paradigm at Time 1 will be positively correlated with samples in the sampling paradigm at Time 2.	Same as Hypothesis 3-1.	Samples in sampling paradigm at Time 1 = the number of samples in each incentivized block prior to final choice, divided by the total number of incentivized blocks at time 1.<br><br>Samples in sampling paradigm at Time 2 = the number of samples in each incentivized block prior to final choice, divided by the total number of incentivized blocks at time 2.	Same as Hypothesis 3-1	Same as Hypothesis 3-1 but with regards to number of samples in the sampling paradigm.	3-6: Hypothesis was confirmed: Number of samples in sampling paradigm at Time 1 was positively correlated with number of samples at Time 2.<br>Question: Number of samples has test-retest reliability.
	Hypothesis 3-7: observe trials in the observe or bet task at Time 1 will be positively correlated with observe trials in the observe or bet task at Time 2.	Same as Hypothesis 3-1.	Observe trials in observe or bet task at Time 1 = number of trials in each incentivized block of the observe or bet task where "observe" is chosen, divided by the total number of incentivized blocks at time 1.<br><br>Observe trials in observe or bet task at Time 2 = number of trials in each incentivized block of the observe or bet task where "observe" is chosen, divided by the total number of incentivized blocks at time 2.	Same as Hypothesis 3-1	Same as Hypothesis 3-1 but with regards to number of observe trials in observe or bet task.	3-7: Hypothesis was confirmed: Number of observe trials in observe or bet task at Time 1 was positively correlated with number of observe trials at Time 2.<br>Question: Number of observe trials has test-retest reliability.

	Hypothesis 3-8: mean self-report ratings on the exploration scale at Time 1 will be positively correlated with mean self-report ratings on the exploration scale at Time 2.	Same as Hypothesis 3-1.	Mean self-report ratings on exploration scale at Time 1.<br><br>Mean self-report ratings on exploration scale at Time 2.	Same as Hypothesis 3-1	Same as Hypothesis 3-1 but with regards to self-report ratings on the exploration scale.	3-8: Hypothesis was confirmed: Ratings on the exploration scale at Time 1 was positively correlated with ratings on the exploration scale at Time 2.<br>Question: The exploration scale has test-retest reliability.
	Hypothesis 3-9: mean self-report ratings on the alternative search subscale at Time 1 will be positively correlated with mean self-report ratings on the alternative search subscale at Time 2.	Same as Hypothesis 3-1.	Mean self-report ratings on alternative search subscale at Time 1.<br><br>Mean self-report ratings on alternative search subscale at Time 2.	Same as Hypothesis 3-1	Same as Hypothesis 3-1 but with regards to self-report ratings on the alternative search subscale.	3-9: Hypothesis was confirmed: Ratings on the alternative search scale at Time 1 was positively correlated with ratings on the alternative search scale at Time 2.<br>Question: The alternative search scale has test-retest reliability.
	Hypothesis 3-10: mean self-report ratings on the maximization tendency scale at Time 1 will be positively correlated with mean self-report ratings on the	Same as Hypothesis 3-1.	Mean self-report ratings on maximization tendency scale at Time 1.<br><br>Mean self-report ratings on maximization tendency scale at Time 2.	Same as Hypothesis 3-1	Same as Hypothesis 3-1 but with regards to self-report ratings on the maximization tendency scale.	3-10: Hypothesis was confirmed: Ratings on the maximization tendency scale at Time 1 was positively correlated with ratings on the maximization tendency scale at Time 2.<br>Question: The maximization tendency

	maximization tendency scale at Time 2.					scale has test-retest reliability.
	Hypothesis 3-11: self-report ratings on the general explore-exploit question at Time 1 will be positively correlated with self-report ratings on the general explore-exploit question at Time 2.	Same as Hypothesis 3-1.	Self-report ratings on general explore-exploit question at Time 1.<br><br>Self-report ratings on general explore-exploit question at Time 2.	Same as Hypothesis 3-1	Same as Hypothesis 3-1 but with regards to self-report ratings on the general explore-exploit question.	3-11: Hypothesis was confirmed: Ratings on the general explore-exploit question at Time 1 was positively correlated with ratings on the general explore-exploit question at Time 2.<br>Question: The general explore-exploit question has test-retest reliability.

References

1. DeGroot, M. H. *Optimal Statistical Decisions.* (John Wiley & Sons, 2005).
2. Gittins, J. C. Bandit Processes and Dynamic Allocation Indices. *J. R. Stat. Soc. Ser. B Methodol.* **41**, 148–164 (1979).
3. Meyer, R. J. & Shi, Y. Sequential choice under ambiguity: Intuitive solutions to the armed-bandit problem. *Manag. Sci.* **41**, 817–834 (1995).
4. Gans, N., Knox, G. & Croson, R. Simple models of discrete choice and their performance in bandit experiments. *Manuf. Serv. Oper. Manag.* **9**, 383–408 (2007).
5. Denrell, J. & March, J. G. Adaptation as information restriction: The Hot Stove Effect. *Organ. Sci.* **12**, 523–538 (2001).
6. Billinger, S., Stieglitz, N. & Schumacher, T. R. Search on rugged landscapes: An experimental study. *Organ. Sci.* **25**, 93–108 (2014).
7. Tracy, W. M., Markovitch, D. G., Peters, L. S., Phani, B. V. & Philip, D. Algorithmic representations of managerial search behavior. *Comput. Econ.* **49**, 343–361 (2017).
8. Vuculescu, O. Searching far away from the lamp-post: An agent-based model. *Strateg. Organ.* **15**, 242–263 (2017).
9. Billinger, S., Srikanth, K., Stieglitz, N. & Schumacher, T. R. Exploration and exploitation in complex search tasks: How feedback influences whether and where human agents search. *Strateg. Manag. J.* **42**, 361–385 (2021).
10. Kauffman, S. A. & Weinberger, E. D. The NK model of rugged fitness landscapes and its application to maturation of the immune response. *J. Theor. Biol.* **141**, 211–245 (1989).
11. Levinthal, D. A. Adaptation on rugged landscapes. *Manag. Sci.* **43**, 934–950 (1997).
12. Telser, L. Searching for the lowest price. *Am. Econ. Rev.* **63**, 40–49 (1973).
13. Sonnemans, J. Strategies of search. *J. Econ. Behav. Organ.* **35**, 309–332 (1998).
14. Hey, J. D. Search for rules for search. *J. Econ. Behav. Organ.* **3**, 65–81 (1982).

15. Hertwig, R., Barron, G., Weber, E. U. & Erev, I. Decisions from experience and the effect of rare events in risky choice. *Psychol. Sci.* **15**, 534–539 (2004).
16. Hertwig, R. & Pleskac, T. J. Decisions from experience: Why small samples? *Cognition* **115**, 225–237 (2010).
17. Tversky, A. & Edwards, W. Information versus reward in binary choices. *J. Exp. Psychol.* **71**, 680–683 (1966).
18. Kashdan, T. B. *et al.* The curiosity and exploration inventory-II: Development, factor structure, and psychometrics. *J. Res. Personal.* **43**, 987–998 (2009).
19. Kashdan, T. B., Rose, P. & Fincham, F. D. Curiosity and exploration: Facilitating positive subjective experiences and personal growth opportunities. *J. Pers. Assess.* **82**, 291–305 (2004).
20. Schwartz, B. *et al.* Maximizing versus satisficing: Happiness is a matter of choice. *J. Pers. Soc. Psychol.* **83**, 1178–1197 (2002).
21. Simon, H. A. A behavioral model of rational choice. *Q. J. Econ.* **69**, 99 (1955).
22. Simon, H. A. Rational choice and the structure of the environment. *Psychol. Rev.* **63**, 129–138 (1956).
23. Highhouse, S. E., Diab, D. L. & Gillespie, M. A. Are maximizers really unhappy? The measurement of maximizing tendency. *Judgm. Decis. Mak. J.* **3**, 364–370 (2008).
24. Nenkov, G. Y., Morrin, M., Ward, A., Schwartz, B. & Hulland, J. A short form of the Maximization Scale: Factor structure, reliability, and validity studies. *Judgm. Decis. Mak.* **3**, 371–388 (2008).
25. Rim, H. B., Turner, B. M., Betz, N. E. & Nygren, T. E. Studies of dimensionality, correlates, and meaning of measures of the maximizing tendency. *Judgm. Decis. Mak.* **6**, 565–579 (2011).
26. Turner, B. M., Rim, H. B., Betz, N. E. & Nygren, T. E. The Maximization Inventory. *Judgm. Decis. Mak.* **7**, 48–60 (2012).

27. Weinhardt, J. M., Morse, B. J., Chimeli, J. & Fisher, J. An Item Response Theory and factor analytic examination of two prominent maximizing tendency scales. *Judgm. Decis. Mak.* **7**, 644–658 (2012).

28. Misuraca, R. & Fasolo, B. Maximizing versus satisficing in the digital age: Disjoint scales and the case for "construct consensus". *Personal. Individ. Differ.* **121**, 152–160 (2018).

29. Harman, J. L., Weinhardt, J. M. & Gonzalez, C. Maximizing scales do not reliably predict maximizing behavior in decisions from experience: Maximizing and DfE. *J. Behav. Decis. Mak.* **31**, 402–414 (2018).

30. Richard S. Sutton & Andrew G. Barto. *Reinforcement Learning: An introduction (adaptive computation and machine learning)*. (MIT Press, 1998).

31. Bhatia, S., He, L., Zhao, W. J. & Analytis, P. P. Cognitive models of optimal sequential search with recall. *Cognition* **210**, 104595 (2021).

32. Soto, C. J. & John, O. P. The next Big Five Inventory (BFI-2): Developing and assessing a hierarchical model with 15 facets to enhance bandwidth, fidelity, and predictive power. *J. Pers. Soc. Psychol.* **113**, 117–143 (2017).
