# Supplementary material for: Testing the convergent validity, domain generality, and temporal stability of selected measures of people’s tendency to explore
Source: Nat Commun. 2024 Sep 4;15:7721. doi: 10.1038/s41467-024-51685-z (PMC11375013; doi:10.1038/s41467-024-51685-z)
Supplement: Supplementary file 3 — Reporting Summary [file 41467_2024_51685_MOESM3_ESM.pdf]

nature portfolio

Corresponding author(s): Farid Anvari; Davide Marchiori

Last updated by author(s): 26/07/2024

Reporting Summary

Nature Portfolio wishes to improve the reproducibility of the work that we publish. This form provides structure for consistency and transparency in reporting. For further information on Nature Portfolio policies, see our Editorial Policies and the Editorial Policy Checklist.

Statistics

For all statistical analyses, confirm that the following items are present in the figure legend, table legend, main text, or Methods section.

n/a	Confirmed	
☐	☒	The exact sample size (*n*) for each experimental group/condition, given as a discrete number and unit of measurement
☐	☒	A statement on whether measurements were taken from distinct samples or whether the same sample was measured repeatedly
☐	☒	The statistical test(s) used AND whether they are one- or two-sided<br>*Only common tests should be described solely by name; describe more complex techniques in the Methods section.*
☒	☐	A description of all covariates tested
☐	☒	A description of any assumptions or corrections, such as tests of normality and adjustment for multiple comparisons
☐	☒	A full description of the statistical parameters including central tendency (e.g. means) or other basic estimates (e.g. regression coefficient) AND variation (e.g. standard deviation) or associated estimates of uncertainty (e.g. confidence intervals)
☐	☒	For null hypothesis testing, the test statistic (e.g. *F*, *t*, *r*) with confidence intervals, effect sizes, degrees of freedom and *P* value noted<br>*Give P values as exact values whenever suitable.*
☒	☐	For Bayesian analysis, information on the choice of priors and Markov chain Monte Carlo settings
☐	☒	For hierarchical and complex designs, identification of the appropriate level for tests and full reporting of outcomes
☐	☒	Estimates of effect sizes (e.g. Cohen's *d*, Pearson's *r*), indicating how they were calculated

Our web collection on statistics for biologists contains articles on many of the points above.

Software and code

Policy information about availability of computer code

Data collection	O-tree software with custom code to run the study and collect data. Participants from Prolific.com
Data analysis	Data analysis done in R with code available at: https://osf.io/f62my/?view_only=98fcafea9c5d468fa938443c63b50381<br>Anonymized data available at: https://osf.io/f62my/?view_only=98fcafea9c5d468fa938443c63b50381

For manuscripts utilizing custom algorithms or software that are central to the research but not yet described in published literature, software must be made available to editors and reviewers. We strongly encourage code deposition in a community repository (e.g. GitHub). See the Nature Portfolio guidelines for submitting code & software for further information.

Data

Policy information about availability of data

All manuscripts must include a data availability statement. This statement should provide the following information, where applicable:

- Accession codes, unique identifiers, or web links for publicly available datasets
- A description of any restrictions on data availability
- For clinical datasets or third party data, please ensure that the statement adheres to our policy

Data analysis done in R with code and data available at: https://osf.io/f62my/?view_only=98fcafea9c5d468fa938443c63b50381

Research involving human participants, their data, or biological material

Policy information about studies with human participants or human data. See also policy information about sex, gender (identity/presentation), and sexual orientation and race, ethnicity and racism.

Reporting on sex and gender	The final sample of participants who completed the full study was 678. These had a mean age of 44 years (SD = 15) ranging from 18 to 82 years, and reported their gender as either female (n = 350), male (n = 323), or other (n = 5).
Reporting on race, ethnicity, or other socially relevant groupings	We recruited a sample from Prolific.com that was matched on age, sex, and gender of the UK population. We do not report any social groupings as these were not relevant for our study.
Population characteristics	We recruited a sample from Prolific.com that was matched on age, sex, and gender of the UK population.
Recruitment	We recruited a sample from Prolific.com that was matched on age, sex, and gender of the UK population.
Ethics oversight	Ethical approval was granted by the Research Ethics Committee of the University of Southern Denmark, case number 19/78419

Note that full information on the approval of the study protocol must also be provided in the manuscript.

Field-specific reporting

Please select the one below that is the best fit for your research. If you are not sure, read the appropriate sections before making your selection.

☐ Life sciences ☒ Behavioural & social sciences ☐ Ecological, evolutionary & environmental sciences

For a reference copy of the document with all sections, see nature.com/documents/nr-reporting-summary-flat.pdf

Behavioural & social sciences study design

All studies must disclose on these points even when the disclosure is negative.

Study description	Quantitative measures of behavioural and self-reported exploration.
Research sample	Adult sample from Prolific.com matched on age, gender, and ethnicity of UK population.
Sampling strategy	Participants recruited from Prolific.com aiming for 95% power to detect r = .2 in one-sided Pearson correlation tests and reject r = .2 in equivalence tests. Required target sample was 632, expecting attrition. Final sample was 678 for Time 1 and 254 for Time 2.
Data collection	Participants recruited from Prolific.com and they completed the study online. Data collected using OTree. Study was conducted online; so only the participants were present.
Timing	Data collection for Time 1 occured April 20th to 28th, 2023; for Time 2 from May 29th to June 2nd, 2023.
Data exclusions	The inclusion/exclusion criteria were preregistered and are fully reported in the manuscript.
Non-participation	n/a
Randomization	n/a

Reporting for specific materials, systems and methods

We require information from authors about some types of materials, experimental systems and methods used in many studies. Here, indicate whether each material, system or method listed is relevant to your study. If you are not sure if a list item applies to your research, read the appropriate section before selecting a response.

Materials & experimental systems

n/a	Involved in the study
☒	☐ Antibodies
☒	☐ Eukaryotic cell lines
☒	☐ Palaeontology and archaeology
☒	☐ Animals and other organisms
☒	☐ Clinical data
☒	☐ Dual use research of concern
☒	☐ Plants

Methods

n/a	Involved in the study
☒	☐ ChIP-seq
☒	☐ Flow cytometry
☒	☐ MRI-based neuroimaging

Plants

Seed stocks	*Report on the source of all seed stocks or other plant material used. If applicable, state the seed stock centre and catalogue number. If plant specimens were collected from the field, describe the collection location, date and sampling procedures.*
Novel plant genotypes	*Describe the methods by which all novel plant genotypes were produced. This includes those generated by transgenic approaches, gene editing, chemical/radiation-based mutagenesis and hybridization. For transgenic lines, describe the transformation method, the number of independent lines analyzed and the generation upon which experiments were performed. For gene-edited lines, describe the editor used, the endogenous sequence targeted for editing, the targeting guide RNA sequence (if applicable) and how the editor was applied.*
Authentication	*Describe any authentication procedures for each seed stock used or novel genotype generated. Describe any experiments used to assess the effect of a mutation and, where applicable, how potential secondary effects (e.g. second site T-DNA insertions, mosiacism, off-target gene editing) were examined.*
